# Magnetic Nanoparticles and Methylprednisolone Based Physico‐Chemical Bifunctional Neural Stem Cells Delivery System for Spinal Cord Injury Repair

**DOI:** 10.1002/advs.202308993

**Published:** 2024-03-22

**Authors:** Wencan Zhang, Mingshan Liu, Jie Ren, Shuwei Han, Xiaolong Zhou, Dapeng Zhang, Xianzheng Guo, Haiwen Feng, Lei Ye, Shiqing Feng, Xizi Song, Lin Jin, Zhijian Wei

**Affiliations:** ^1^ Department of Orthopaedics Qilu Hospital of Shandong University Shandong University Centre for Orthopaedics Advanced Medical Research Institute Shandong University No. 107 Wenhua West Road, Lixia District Jinan 250012 China; ^2^ Department of Orthopedics Tianjin Medical University General Hospital International Science and Technology Cooperation Base of Spinal Cord Injury Tianjin Key Laboratory of Spine and Spinal Cord Injury No. 154 Anshan Road, Heping District Tianjin 300052 China; ^3^ NMPA Key Laboratory for Technology Research and Evaluation of Drug Products and Key Laboratory of Chemical Biology (Ministry of Education) Department of Pharmaceutics School of Pharmaceutical Sciences Cheeloo College of Medicine Shandong University No. 44 Wenhua West Road, Lixia District Jinan 250012 China; ^4^ Academy of Medical Engineering and Translational Medicine Tianjin University No. 92 Weijin Road, Nankai District Tianjin 300072 China; ^5^ International Joint Research Laboratory for Biomedical Nanomaterials of Henan Zhoukou Normal University No. 6, Middle Section of Wenchang Avenue, Chuanhui District Zhoukou 466001 China

**Keywords:** functional neurogenesis, magnetic nanoparticles guidance, mechanical stimulation, neural stem cell transplantation, spinal cord injury repair

## Abstract

Neural stem cells (NSCs) transplantation is an attractive and promising treatment strategy for spinal cord injury (SCI). Various pathological processes including the severe inflammatory cascade and difficulty in stable proliferation and differentiation of NSCs limit its application and translation. Here, a novel physico‐chemical bifunctional neural stem cells delivery system containing magnetic nanoparticles (MNPs and methylprednisolone (MP) is designed to repair SCI, the former regulates NSCs differentiation through magnetic mechanical stimulation in the chronic phase, while the latter alleviates inflammatory response in the acute phase. The delivery system releases MP to promote microglial M2 polarization, inhibit M1 polarization, and reduce neuronal apoptosis. Meanwhile, NSCs tend to differentiate into functional neurons with magnetic mechanical stimulation generated by MNPs in the static magnetic field, which is related to the activation of the PI3K/AKT/mTOR pathway. SCI mice achieve better functional recovery after receiving NSCs transplantation via physico‐chemical bifunctional delivery system, which has milder inflammation, higher number of M2 microglia, more functional neurons, and axonal regeneration. Together, this bifunctional NSCs delivery system combined physical mechanical stimulation and chemical drug therapy is demonstrated to be effective, which provides new treatment insights into clinical transformation of SCI repair.

## Graphical abstract

A novel physico‐chemical bifunctional NSCs delivery system containing MNPs, and MP is designed to repair SCI targeting multiple pathological processes by promoting microglial M2 polarization and reducing neuronal apoptosis in the acute phase, enhancing functional neurons through magnetic mechanical stimulation in the chronic phase related to the PI3K/AKT/mTOR pathway. This bifunctional NSCs delivery system provides new treatment insights into clinical transformation.

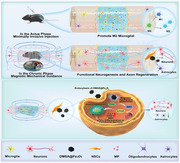

## 1 Introduction

Spinal cord injury (SCI) is a severe traumatic disease with high disability and mortality due to various pathological processes and a lack of adequate clinical treatment strategies.^[^ ^1^ ^]^ After spinal cord injury, a large number of inflammatory chemokines and cytokines are released locally, damaging the surrounding tissue and cells and forming an inflammatory microenvironment, continuously exacerbating the damage.^[^ ^2^ ^]^ Differentiation of endogenous neural stem cells (NSCs) into neurons has a positive effect on SCI repair, however, they are insufficient at the injury site. Moreover, the inflammatory microenvironment makes endogenous NSCs difficult to stably increase in vivo, and it is more inclined to differentiate into astrocytes, producing glial scars and hindering nerve regeneration and the reconstruction of the neural circuit.^[^ ^3^, ^4^ ^]^

NSCs transplantation can solve the problem of insufficient endogenous NSCs after SCI and is a promising treatment strategy.^[^ ^5^ ^]^ However, this approach also faces some challenges, including low survival rates, difficulty in stable proliferation and differentiation at the injury site, and inability to repair large spinal cord defects.^[^ ^6^ ^]^ There is an urgent need to develop NSCs delivery system improving the inhibitory microenvironment after SCI and ensure the stable proliferation and directional differentiation of NSCs into neurons.^[^ ^7^, ^8^ ^]^

Hydrogel can simulate the physiological state of the extracellular matrix, which is conducive to the adhesion, expansion, and differentiation of NSCs at the injury site, and can fill the cavity in a structural replacement way, thus promoting nerve regeneration and functional recovery.^[^ ^9^ ^]^ Inflammatory response is one of the essential components of secondary injury after SCI, making NSCs difficult to stably proliferate in vivo and differentiate toward neurons.^[^ ^10^ ^]^ Methylprednisolone (MP) is a corticosteroid which was currently recommended by European medical institutions and the Food and Drug Administration for SCI.^[^ ^11^ ^]^ However, the high‐dose and long‐term systemic administration can lead to serious complications, including increasing the risk of urinary tract, respiratory tract, and wound infections, significantly limiting its clinical application.^[^ ^12^ ^]^ The 3D pore structure of hydrogel makes it have good performance in drug release. Hydrogel can be used to achieve local release of MP, which effectively avoids the problems caused by systemic application.^[^ ^13^ ^]^

The reinnervation and functional recovery after SCI depend on the number of neurons and the longitudinal directional regeneration of axons.^[^ ^14^ ^]^ Without guidance, NSCs after transplantation are difficult to differentiate mostly into functional neurons.^[^ ^15^ ^]^ Moreover, the direction of axonal regeneration is often disrupted, which limits the ability to form a complete relay signal station, significantly increasing the difficulty of treating SCI.^[^ ^16^ ^]^ With the rapid development of biomedicine, magnetic stimulation technology has been proven to play a positive role in stem cell proliferation and differentiation.^[^ ^17^ ^]^ NSCs endocytosis of magnetic nanoparticles (MNPs) at the injury site makes it possible to obtain guidance from external magnetic stimulation. DMSA coated Fe_3_O_4_ nanoparticles (DMSA@Fe_3_O_4_) have magnetic responsiveness, which can be regulated by an external magnetic field as a component of the hydrogel. At the same time, it has superparamagnetism, which can avoid the magnetocaloric effect in the static magnetic field (SMF).

**Figure 1:**
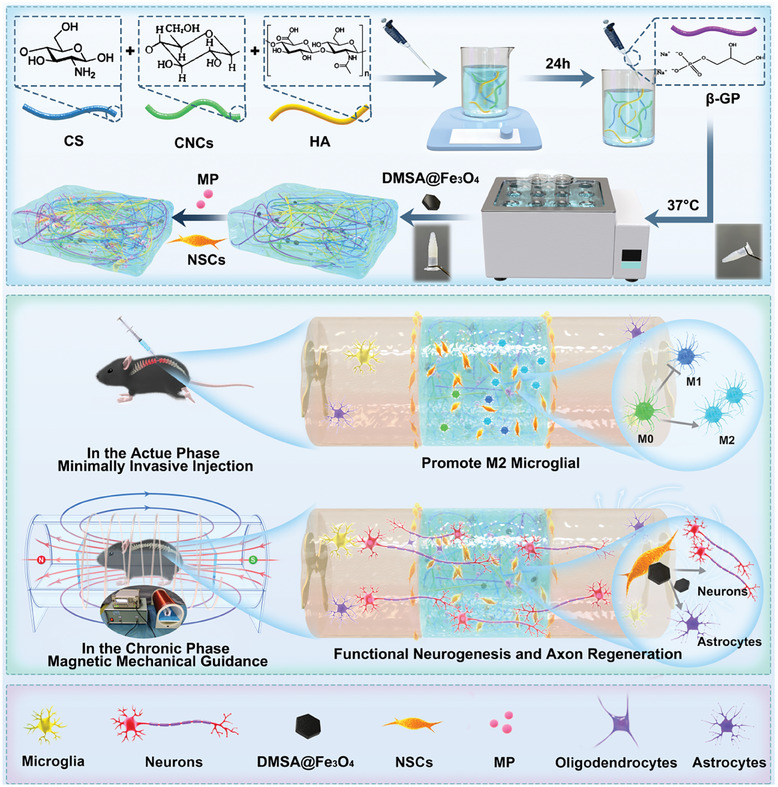
The diagram of the design and fabrication of delivery system loading DMSA@Fe_3_O_4_ and MP for the repair of spinal cord injury. A novel magnetic and temperature‐responsive nanohydrogel was synthesized as a suitable delivery system for NSCs transplantation and MP sustained release, which could be minimally invasive injected. This delivery system alleviated the inflammatory microenvironment in the acute phase and promoted functional neurogenesis and axon regeneration with the static magnetic guidance in the chronic phase.

The present study develops a bifunctional NSCs delivery system based on a temperature‐responsive nanohydrogel that contains DMSA@Fe_3_O_4_ as the regulator of the physical mechanical stimulation and MP for the chemical drug therapy. In the acute phase, the delivery system can release MP to promote microglial M2 polarization, inhibit M1 polarization, and reduce neuronal apoptosis. In the chronic phase, NSCs tend to differentiate into functional neurons in better microenvironment with magnetic mechanical stimulation generated by magnetic nanoparticles in the SMF (**Figure** **1**). This bifunctional NSCs delivery system combined physical mechanical stimulation and chemical drug therapy is demonstrated to be effective, which provides new treatment insights into clinical transformation of SCI repair.

## 2 Results

### 2.1 Fabrication and Characterizations of Hydrogel

**Figure 2:**
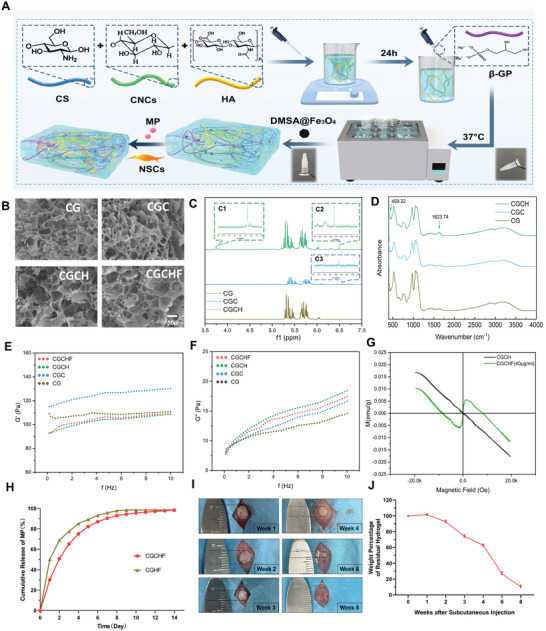
Synthesis and characterizations of hydrogel. A) the multistep synthesis process was performed to produce CGCHF hydrogels. B) SEM analysis showed that CG, CGC, CGCH, and CGCHF exhibited an excellent 3D structure. C) The 1H NMR result of CGCH hydrogel. D) The FTIR results of CGCH hydrogel. E,F) Rheological analysis of the hydrogel. G) The CGCHF hydrogel with 40 ug mL^−1^ DMSA@Fe_3_O_4_ had obvious magnetic responsiveness compared with CGCH hydrogel. H) The release of MP in the CGCHF group within 14 days. I,J) The degradation of CGCHF hydrogel within 8 weeks (*n* = 3).

A multistep synthesis process was performed to produce CGCHF hydrogel (**Figure** **2**A). First, the CG hydrogel was formed by 50% β‐Glycerol phosphate disodium salt (β‐GP) solution and 2% Chitosan (CS) solution with a certain volume ratio 1:5 at 37 °C. Then, Cellulose nanocrystals (CNCs) were used as a structural stabilizer of hydrogel to add into 2% CS solution, and 30% β‐GP solution was added to form CGC nanohydrogel, reducing the concentration of β‐GP and enhancing biocompatibility. Hyaluronic acid (HA) was added as a structural component of hydrogel to promote the attachment of NSCs and improve the biocompatibility of CGCH hydrogel. Due to magnetic responsiveness, DMSA coated Fe_3_O_4_ nanoparticles (DMSA@Fe_3_O_4_) were added as a component of CGCHF hydrogel for endocytosis of NSCs under the guidance of SMF.

SEM images of CGC hydrogel showed a satisfactory 3D porous structure for the proliferation and differentiation of cells. (Figure S1A, Supporting Information). For injectable thermosensitive hydrogels, faster gel time is significant for clinical transformation. CGC hydrogel of 10% CNC can be gel within 6 min under 37 °C in vitro and has good water content (Figure S1B,C, Supporting Information). Rheological analysis showed that the storage moduli (G′) and the loss moduli (G″) of CGC hydrogel with 10% CNC were similar to that of CG hydrogel while reducing GP concentration (Figure S1E,F, Supporting Information). Therefore, 10% CNC was considered the optimal addition ratio to reduce GP concentration. Meanwhile, according to the gel time, 4% HA was selected to enhance the biocompatibility (Figure S1B,C, Supporting Information). The result of 1H NMR showed that CGCH hydrogel has a unique characteristic peak at 6.5 ppm for CNCs (Figure 2C2,C3; Figure S2A2, Supporting Information) and 3.2–3.5 ppm for HA (Figure 2C1; Figure S2A1, Supporting Information). The results of FTIR showed that the CGCH hydrogel exhibited characteristics of CG. The peaks at 1623.74 and 409.22 cm^−1^ are CNCs and HA vibrations, which appear in the spectrum of CGCH hydrogel, indicating that CNC and HA are successfully combined into the CG hydrogel skeleton (Figure 2D). As a result, CGCH hydrogel was synthesized, which can be quickly gel at 37 °C in vitro (Figure S1D, Supporting Information). And it can be injected subcutaneously into C57 mice to form a hydrogel in situ (Figure S1G, Supporting Information). Meanwhile, Rheological analysis showed that the shear thinning and temperature‐responsive ability to establish the hydrogel's minimally invasive injection capability (Figure S1H,I, Supporting Information).

The concentration of DMSA@Fe_3_O_4_ was determined based on its magnetic responsiveness and biological toxicity. SEM analysis displayed the morphology of DMSA@Fe_3_O_4,_ with the majority having a diameter of 10 nm (Figure S3A,B, Supporting Information), which can be attracted by a cylindrical magnet (Figure S3C, Supporting Information). The result of the hysteresis loop indicated that its magnetic responsiveness gradually increased as the concentration of DMSA@Fe_3_O_4_ increased (Figure S3D, Supporting Information). After extracting primary neural stem cells, immunofluorescence was used to identify their markers such as SOX2 and Nestin (Figure S4A, Supporting Information). The CCK8 results showed that DMSA@Fe_3_O_4_ has a certain dose‐dependent effect on the proliferation of NSCs. However, the impact of promoting proliferation became weak when the concentration of DMSA@Fe_3_O_4_ reached 80ug mL^−1^ Therefore, 40ug mL^−1^ of DMSA@Fe_3_O_4_ was adopted to the hydrogel. Prussian blue staining results show that NSCs can absorb more DMSA@Fe_3_O_4_ at a concentration of 40ug mL^−1^ (Figure S4C, Supporting Information).

SEM analysis showed that CG, CGC, CGCH, and CGCHF exhibited a good 3D structure (Figure 2B). Rheological analysis results showed that adding HA and DMSA@Fe_3_O_4_ did not significantly change the G′ and G″ of the hydrogels. On the contrary, the mechanical properties of CGCHF were closer to the elastic modulus of neural tissue (Figure 2E,F). The magnetic responsiveness of CGCHF hydrogel was positively related to the concentration of DMSA@Fe_3_O_4_ (Figure S2B, Supporting Information). The CGCHF hydrogel with 40 ug mL^−1^ DMSA@Fe_3_O_4_ had apparent magnetic responsiveness compared with CGCH hydrogel (Figure 2G). The water content of CGCHF was not significantly different from CG, CGC, and CGCH (Figure S2C, Supporting Information). And the gel time of CGCHF was consistent with CGCH (Figure S2D, Supporting Information). HLPC was used to analyze the rate of MP release, which showed that the sustained release of MP in the CGCHF group was slower than that of hydrogel without CNCs within 14 days (Figure 2H), which was beneficial for the more sustained release of MP during the acute phase (within 3 days) and subacute phase (within 14 days) of SCI. CGCHF hydrogel was injected under the back skin of the C57 mice to evaluate its degradation in vivo. The results showed that CGCHF hydrogel could gradually degrade within 8 weeks (Figure 2I,J).

### 2.2 Biocompatibility and Biotoxicity of Hydrogel

After NSCs were co‐cultured with the hydrogel for 96 h, survival, and proliferation were detected by live/dead staining at different time points. The number of NSCs on the CG hydrogel decreased significantly at each time point, while the number of NSCs on the CGC hydrogel increased, which was related to the lower β‐GP concentration. After adding HA and DMSA@Fe_3_O_4_, the proliferation of NSCs was further improved (Figure S5A,B, Supporting Information). SEM results showed that NSCs could adhere and proliferate well on the surface and inside CGCHF hydrogel (Figure S6A,B, Supporting Information). These results indicated that adding CNCs, HA, and FE can improve the biocompatibility of hydrogel. The hydrogels were subcutaneously injected into the back of C57 mice, and the subcutaneous inflammatory reaction was detected 1 week later. HE staining showed that 1 week after the implantation of the hydrogel, neutrophils infiltrated subcutaneously around the hydrogel, and immunofluorescence results showed that the intensity of CD68 (macrophage marker) was lower in CGCHF hydrogel (Figure S7A,B, Supporting Information). At the same time, histological analysis confirmed that the main organs (heart, liver, lung, and kidney) of mice in each group had no noticeable pathological changes (Figure S8A, Supporting Information). The levels of important liver and kidney function indicators such as ALT, AST, ALP, CERA, BUN, and UA in SCI mice treated with different treatments after 8 weeks were within the normal range, indicating that the multiple combination therapy proposed in this study did not cause systemic toxicity in SCI mice (Figure S8B, Supporting Information).

### 2.3 Delivery System Containing MP Inhibited Inflammation, Promoted M2 Polarization, and Reduced Neuronal Apoptosis In Vitro

**Figure 3:**
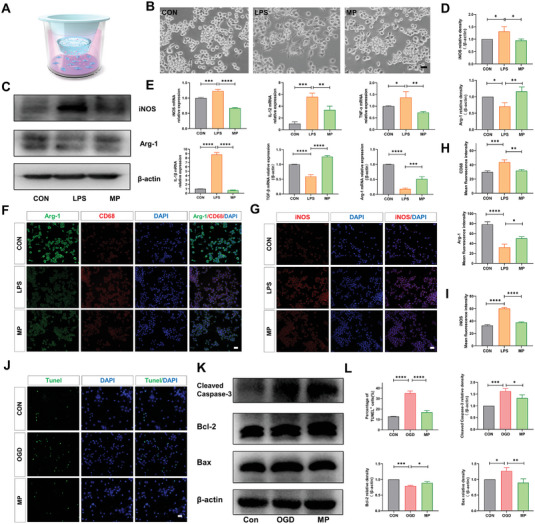
CGCH hydrogel loaded with MP inhibited inflammation, promoted M2 polarization, and reduced neuronal apoptosis in vitro. A) The co‐culture system of hydrogel and BV2. B) BV2 inflammation model. Scale bar: 100 µm C,D) Representative western blots. (*n* = 3). E) The PCR results (*n* = 3). F,H) Representative immunofluorescence images and analysis results of CD68 positive and Arg‐1 positive cells (*n* = 5). Scale bar: 50 µm. G,I) Immunofluorescence images of iNOS positive cells and its analysis results (*n* = 5). Scale bar: 50 µm. J,L) The Tunel staining images and analysis results of primary neurons (*n* = 5). Scale bar: 50 µm. K,L) Representative western blots of apoptosis‐associated proteins. (*n* = 3). ^*^ *p* < 0.05, ^**^ *p* < 0.01, ^***^ *p* < 0.001.

A co‐culture system of CGCHF hydrogel and microglia (BV2) was established by using transwell cells, where BV2 cells were cultured in the lower layer, and MP loaded CGCHF hydrogel was placed on the upper layer to simulate the drug release process (**Figure** **3**A). BV2 inflammation model has been established, and LPS can induce BV2 cells to exhibit amoeba morphology, which can be suppressed after MP treatment (Figure 3B). Microglia tends to divide into M1 phenotype (pro‐inflammatory) whose markers mainly include iNOS, IL‐12, TNF‐α, and IL‐1β, but M2 phenotype (anti‐inflammatory) whose markers mainly include Arg‐1 and TGF‐ β after SCI. The WB results showed that the CGCHF hydrogel loaded with MP promoted Arg‐1 expression and inhibited iNOS expression (Figure 3C,D). The PCR results showed that the RNA expression of iNOS, IL‐12, IL‐1 β, and TNF‐α was decreased in the MP treatment group, while the RNA expression of Arg‐1 and TGF‐β was significantly increased (Figure 3E). The immunofluorescence results of CD68 and iNOS showed a significant decrease in the MP treatment group. Meanwhile, the intensity of arg‐1 increased (Figure 2F,G,H,I). These findings proved that the CGCHF hydrogel loaded with MP can inhibit the inflammatory response and promote M2 polarization of BV2 cells. Furthermore, in order to demonstrated its protective effect on neurons, primary neurons were extracted and an OGD (oxygen‐glucose deprivation/reoxygenation) model of neurons was established to simulate ischemia of neural cells after SCI. The Tunel staining results showed that pretreatment of CGCHF hydrogel loaded with MP could reduce the number of neuronal apoptosis (Figure 3J,L), and the WB results further confirmed that it reduced cleaved‐caspase 3 and bax (pro‐apoptotic proteins) caused by OGD, while increasing bcl‐2 (anti‐apoptotic protein) (Figure 3K,L).

### 2.4 NSCs Tended to Differentiate into Functional Neurons with Magnetic Guidance

**Figure 4:**
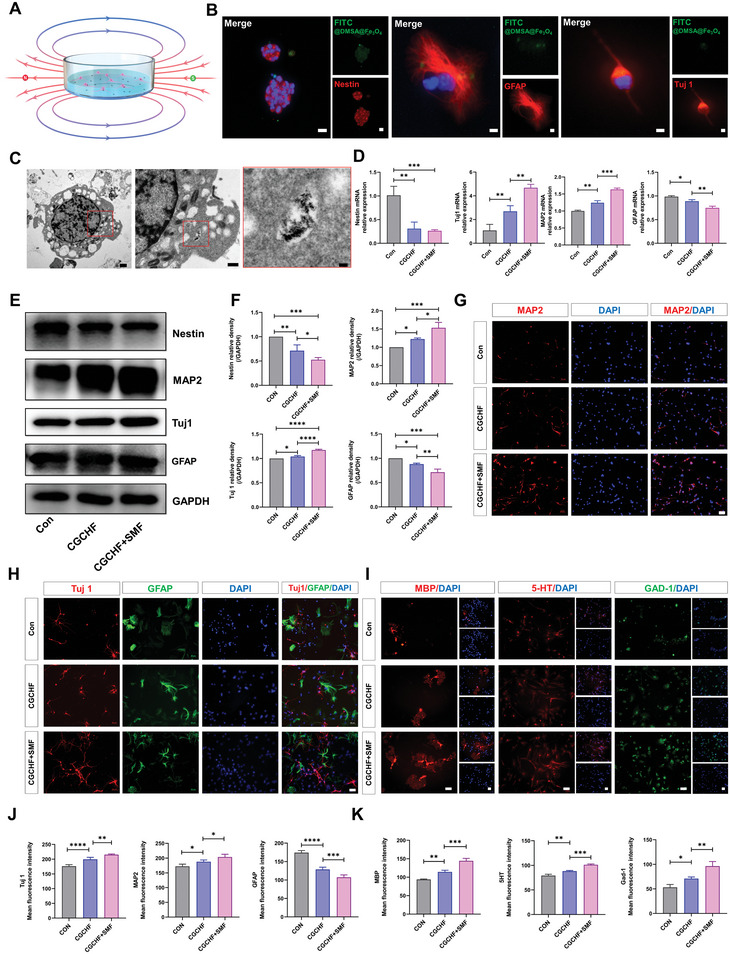
NSCs tended to differentiate into functional neurons after endocytosis of DMSA@Fe_3_O_4_ under the guidance of SMF. A) NSCs co‐cultured with CGCHF hydrogel and placed in SMF. B) Representative images of FITC‐labeled DMSA@Fe_3_O_4_ internalized into the Nestin positive neurosphere(Scale bar:  20 µm) and astrocytes, neurons (Scale bar: 100 µm). C) TEM images of nanoparticles in NSCs differentiated cells. Scale bars: 1, 0.5, and 0.1 µm. D) The PCR results of the level gene expression of nestin (*n* = 3). E,F) Representative western blots s. (*n* = 3). G,J) Representative immunofluorescence images and analysis results of MAP2 positive cells. (*n* = 5). Scale bar: 50 µm. H,J) Representative immunofluorescence images and analysis results of Tuj 1 positive and GFAP positive cells. (*n* = 5). Scale bar: 50 µm. I,K) Representative immunofluorescence images and analysis results of MBP positive, 5‐HT positive, and GAD‐1 positive cells. Scale bar: 50 µm. ^*^ *p *< 0.05, ^**^ *p *< 0.01, ^***^ *p *< 0.001.

NSCs were co‐cultured with CGCHF hydrogel and placed in SMF to explore their differentiation (**Figure** **4**A). The immunofluorescence results confirmed that the DMSA@Fe_3_O_4_ nanoparticles were internalized into the neurosphere and remained after being labeled with FITC. Even when NSCs differentiated into neurons and astrocytes, the co‐localization of nanoparticles and cells could be observed (Figure 4B; Figure S3E, Supporting Information). It can also be observed using TEM that nanoparticles exist in NSCs differentiated cells, mainly in the cytoplasm (Figure 4C). The PCR results showed that Nestin in the CGCHF+SMF group was reduced compared with the control group and CGCHF group, indicating that NSCs had more differentiation, promoted gene expression of immature neuron marker (Tuj 1) and mature neuron marker (MAP2), and inhibited gene expression of astrocyte marker (GFAP) (Figure 4D). The WB results also showed that after implantation of SMF, CHCHF hydrogel significantly increased the expression of Tuj1 and MAP2 after NSC differentiation while reducing the expression of GFAP (Figure 4E,F). The immunofluorescence results displayed that after implantation of SMF, CHCHF hydrogel not only significantly increased the expression of Tuj1 and MAP2 after NSCs differentiation but also enhanced the expression level of oligodendrocyte marker (MBP) and depressed the GFAP (Figure 4G,H,I,J). In order to further investigate the type of neurons derived from NSCs differentiation, immunofluorescence staining was carried out for the 5‐hydroxytryptaminergic neurons marker (5HT) and GABAergic neurons marker (GAD‐1). The number of 5HT and GAD‐1 positive cells in the CGCHF group was apparently elevated, which became more numerous after SMF treatment (Figure 4I,K). These results indicated that after endocytosis of DMSA@Fe_3_O_4,_ NSCs can differentiate more into immature neurons, mature neurons, oligodendrocytes, and less into astrocytes under the guidance of SMF, which also improved the number of 5‐hydroxytryptaminergic neurons and GABAergic neurons.

### 2.5 The Mechanism of Mechanical Stimulation Regulating NSCs through DMSA@Fe_3_O_4_

**Figure 5:**
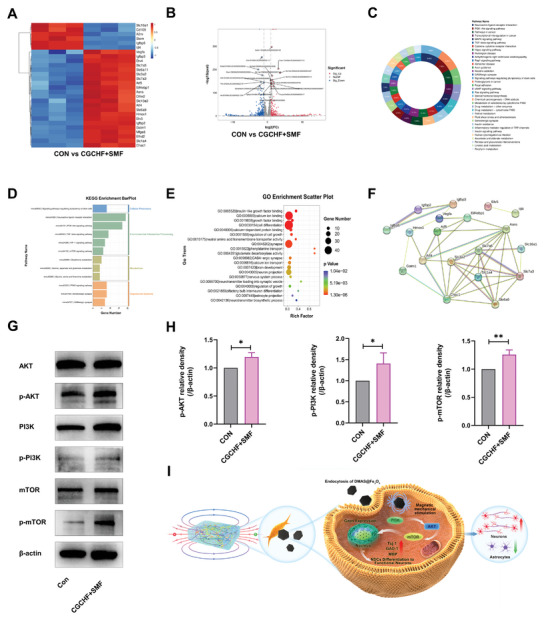
The mechanism of SMF regulating NSCs through DMSA@Fe_3_O_4_. A) The RNA seq results showed 28 genes with significant differences in neuronal development and protection, neural inhibitory, and axon extension and guidance, myelin regeneration and synaptic structure (*n* = 3). B) The gene volcano map of Top20 shows that Gstm1 and Chac1 are most significantly elevated (*n* = 3). C) The doughnut diagram of the KEGG pathway (*n* = 3). D) The bar chart of KEEG enrichment shows PI3K/AKT signaling pathway, mTOR signaling pathway (*n* = 3). E) The bubble diagram of GO enrichment (*n* = 3). F) Protein interaction network (n=3). G) Representative western blots showing the expression of PI3K, p‐PI3K, AKT, p‐AKT, mTOR, and p‐mTOR. H) Quantitative analysis of the p‐PI3K/β‐actin ratio, p‐AKT/β‐actin ratio, and p‐mTOR/β‐actin ratio (*n* = 3). I) Schematic diagram of the mechnism. Significance: ^*^ *p* < 0.05, ^**^ *p* < 0.01.

In order to investigate the mechanism of SMF on NSCs differentiation after endocytosis of DMSA@Fe_3_O_4_, RNA seq analysis was performed. Twenty‐eight genes with significant differences were screened out that related to neuronal development and protection (Slc7a5, Slc6a11, Slc3a2, Slc7a3, Slc1a4, Slc6a9, Chac1, Slc13a3, Eif4ebp1, Atf5, Atf4, Hmox1, Igfbp2, Igfbp3, Mfge8, Etv5, Asns), neural inhibitory (A2m, Igfbp5, Stom, Id4, Slc16a1, Cd109), and axon extension and guidance (Efhd2, Efbp3, Mfge8, Etv5, Asns). tv4, Vegfa), myelin regeneration (Gstm1), synaptic structure (Olfm2) (**Figure** **5**A). The gene volcano map of Top20 shows that Gstm1 and Chac1 are most significantly elevated (Figure 5B). The doughnut diagram shows the KEGG pathway (Figure 5C). The bar chart of KEEG enrichment shows that it can be enriched in the neuroactive ligand receiver interaction, PI3K/AKTsignaling pathway, mTOR signaling pathway, serotonergic synapse, and GABAergic synapse (Figure 5D). The bubble diagram of GO enrichment shows that it can be enriched in calcium ion binding, cell differentiation, synapse, GABA electric synapse, and neuron projection (Figure 5E). The protein interaction network showed the connections between proteins related to the twenty‐eight genes (Figure 5F). According to the sequencing results, PI3K/AKT/mTOR signaling pathway was considered to have a certain connection with NSCs differentiation. Furthermore, the WB results indicated that PI3K, p‐PI3K, AKT, p‐AKT, mTOR, and p‐mTOR were higher in the CGCHF+SMF group (Figure 5G,H). This result indicated that the mechanism of SMF regulating NSCs through DMSA@Fe_3_O_4_ had relevance to the activation of the PI3K/AKT/mTOR pathway (Figure 5I).

### 2.6 The Delivery System Loaded with MP and NSCs Combined with SMF Treatment Improved Functional Recovery After SCI

**Figure 6:**
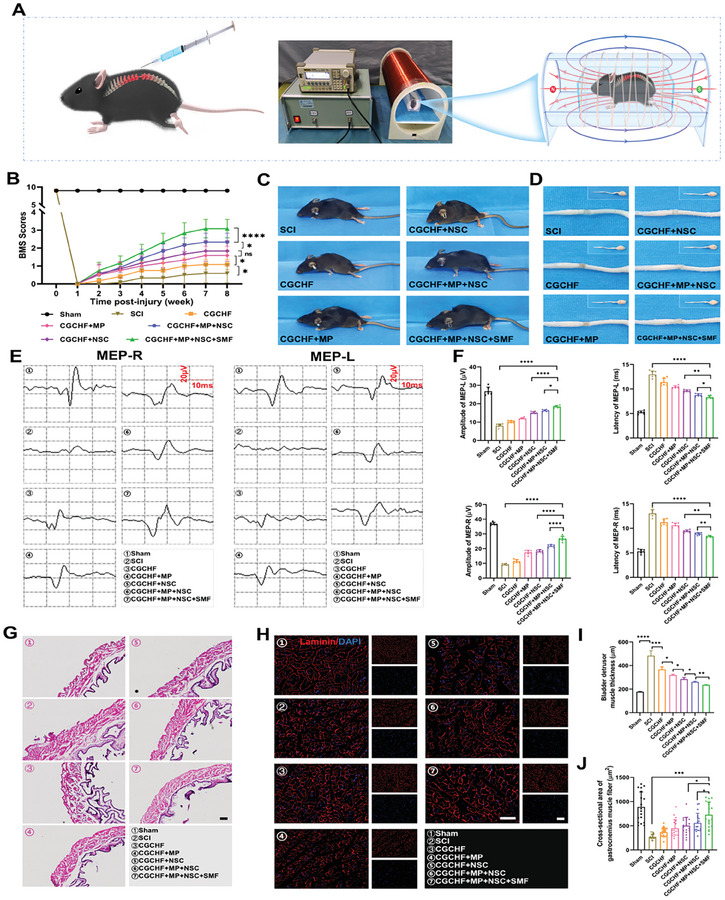
The delivery system loaded with MP and NSCs combined with SMF treatment improved functional recovery after SCI. A) The scheme of the delivery system injected into fill the defect and received SMF treatment. B) The BMS score of spinal cord transection mice treated with several different methods. (n=6). C) The morphology of the hind limbs during walking in each group after 8 weeks of treatment. D) The morphology and color of the tissue at the SCI defect site at 8 weeks after SCI. E,F) Representative electrophysiological images and Quantitative analysis of MEP‐R and MEP‐L signals in each group(n=6). G,I) Representative images of HE staining of the bladder detrusor muscle and quantitative analysis of the thickness (n=6). Scale bar: 200 µm. H) Representative images of laminin staining on the gastrocnemius muscles to mark the area of muscle fibers. Scale bar: 100 µm. J) Quantitative analysis of the cross‐sectional area of gastrocnemius muscle fibers in the hind limbs of mice in each group*(n* = 18, six animals per group with three muscle fibers counted for each animal). ^*^ *p *< 0.05, ^**^ *p *< 0.01, ^***^ *p *< 0.001.

After establishing the SCI transection model, the delivery system was injected to fill the defect and received SMF treatment for 2 h per day (**Figure** **6**A). The functional recovery of mice was evaluated using the BMS score. In the spinal cord transection model, the animal immediately experienced complete paralysis of the hind limbs (0 score) (Figure 6B). The BMS scores of mice in different treatment groups showed varying degrees of increase. And the lower limb function of mice restored to varying degrees (Figure 6C). These findings firmly declared that the delivery system carried with MP and NSCs combined with SMF significantly improves functional recovery after SCI.

The electrophysiological experiments revealed that the amplitude of MEP‐R and MEP‐L signals recorded in the CGCHF hydrogel combined with the MP and NSCs treatment group was evidently higher than that of other single treatment groups (Figure 6E,F). After SMF treatment, the amplitude of MEP‐R and MEP‐L signals was further improved (Figure 6E,F). These findings further demonstrated that the CGCHF hydrogel loaded with MP and NSCs combined with SMF therapy was superior to other treatment methods. At 8 weeks after SCI, the spinal cord was dissected and it was found that the CGCHF hydrogel could effectively fill the defect. After transplantation of NSCs, the morphology and color of the tissue at the defect site were similar to normal spinal cord tissue, especially in the CGCHF hydrogel loaded with MP and NSCs combined with the SMF treatment group (Figure 6D). The thickness of the bladder detrusor muscle in SCI mice increases in a reactive manner, which is related to the stimulation of inflammation in the bladder and the need for stronger muscle strength for involuntary urination. HE staining of the bladder detrusor muscle revealed that the detrusor muscle in the CGCHF hydrogel loaded with MP and NSCs combined with the SMF treatment group was thinner than the other therapy group and close to that in the sham group (Figure 6G,I). Laminin staining was performed on the gastrocnemius muscles to mark the area of muscle fibers and evaluate the atrophy extent. After SCI, the cross‐sectional area of gastrocnemius muscle fibers in the hind limbs of mice was significantly reduced due to atrophy. After treatment, the cross‐sectional area of muscle fibers was improved, further proving that the CGCHF hydrogel loaded with MP and NSCs combined with SMF treatment could improve the muscle motor function of the hind limbs after SCI. (Figures 6H,J).

### 2.7 The Delivery System Loaded with MP Regulated Inflammation in SCI Mice

**Figure 7:**
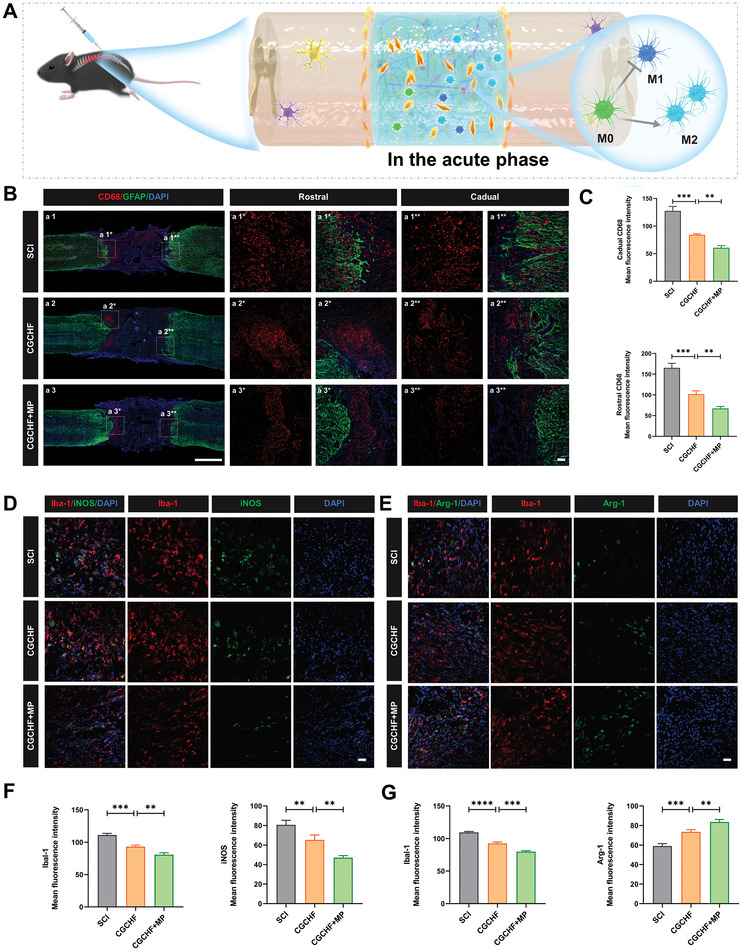
The delivery system loaded with MP regulated inflammation three days after SCI. A) The mechanism of the delivery system loaded with MP to inhibit the inflammatory response in the acute phase after SCI. B) Representative immunofluorescence images of CD68 and GFAP on day three after SCI. Scale bar: 1000 and 50 µm. C) Quantitative analysis of the number of CD68 positive cells in the rostral and caudal(*n* = 3). D) Representative immunofluorescence images of Ibal‐1 and iNOS on day three after SCI. Scale bar: 50 µm. E) Representative immunofluorescence images of Ibal‐1 and Arg‐1 on day three after SCI (*n* = 3). Scale bar: 50 µm. F) Quantitative analysis of Ibal‐1 and iNOS positive cells (*n* = 3). G) Quantitative analysis of the number of Ibal‐1 and Arg‐1 positive cells (n = 3). ^*^ *p *< 0.05, ^**^ *p *< 0.01, ^***^ *p *< 0.001.

Inhibiting inflammatory response and weakening the degree of secondary injury after SCI can provide a favorable microenvironment for the repair of SCI. In the SCI group, the higher intensity of CD68 at the injury site suggested that severe inflammation exited three days after SCI (**Figure** **7**B). After treatment with the CGCHF hydrogel, the CD68 intensity at both ends of the injury can be reduced while the CGCHF hydrogel with MP further reduced the CD68 intensity, indicating that the CGCHF hydrogel loaded with MP significantly inhibited the inflammatory response at the injury site (Figure 7B,C). Immunofluorescence staining of Ibal‐1 (macrophage/microglia marker) and iNOS was performed to figure out the M1 polarization. The results showed a significant decrease in Ibal‐1 and iNOS positive cells in the MP loaded CGCHF hydrogel group (Figure 7D,F). Immunofluorescence staining of Ibal‐1 and Arg‐1 was performed to probe into the M2 polarization, indicating a significant decrease in Ibal‐1 positive cells and an increase in Arg‐1 positive cells in the MP loaded CGCHF hydrogel group (Figure 7E,G). The above findings demonstrated that the CGCHF hydrogel carried MP early inhibited the inflammatory response after SCI, promoted M2 polarization, and inhibited M1 polarization, thereby improving the immune microenvironment and playing a positive role in SCI repair (Figure 7A).

### 2.8 Magnetic Guidance Enhanced Neurogenesis, Axonal Regeneration, Remyelination, and Functional Neurons In Vivo

**Figure 8:**
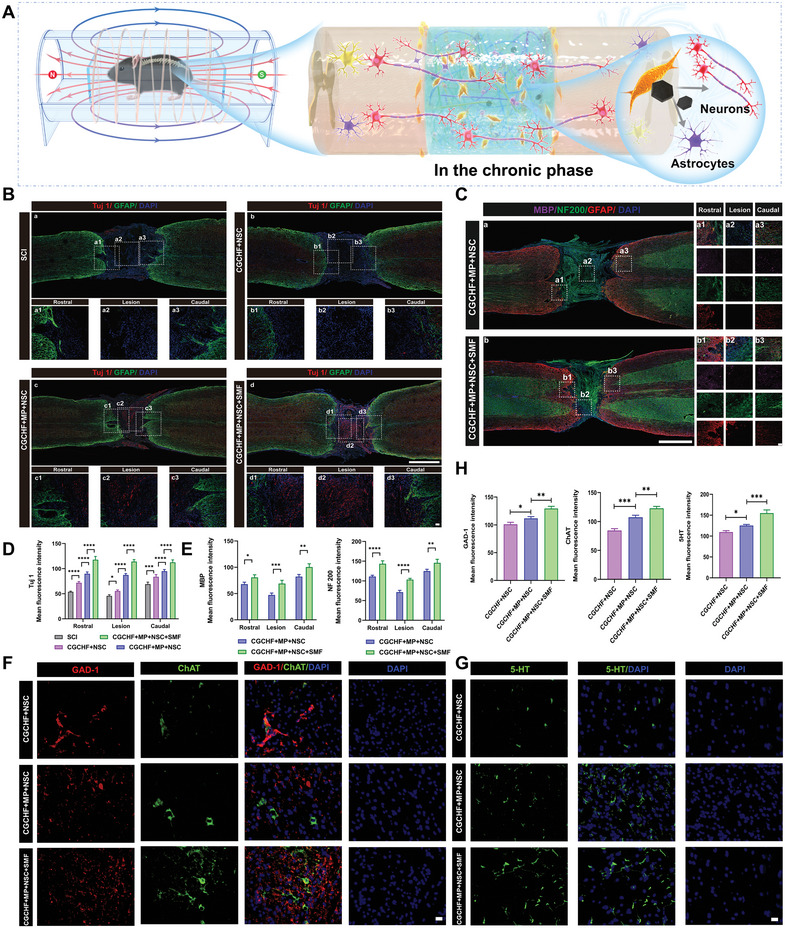
SMF guidance enhanced neurogenesis, axonal regeneration, remyelination, and functional neurons 8 weeks after SCI. A) The repair mechanism under the regulation of SMF in the chronic phase. B) Representative immunofluorescence images of Tuj 1 and GFAP. Scale bar: 1000 and 50 µm. C) Representative immunofluorescence images of MBP, NF200, and GFAP. Scale bar: 1000 and 50 µm. D) Quantitative analysis of the number of Tuj 1 positive cells in the rostral, lesion, and caudal.(n=3) E) Quantitative analysis of the number of MBP and NF200 positive cells in the rostral, lesion, and caudal (n=3). F) Representative immunofluorescence images of GAD‐1 and ChAT at the lesion site. Scale bar: 20 µm. G) Representative immunofluorescence images of 5‐HT at the lesion site. Scale bar: 20 µm. H) Quantitative analysis of the number of GAD‐1, ChAT, and 5‐HT positive cells in the lesion (n=3). ^*^ *p *< 0.05, ^**^ *p *< 0.01, ^***^ *p *< 0.001.

The number of neurons and axonal connections in the chronic phase are crucial for the recovery of SCI. In order to further investigate thoroughly the functional recovery mechanism related to NSCs differentiation and neuronal types, histological analysis of the spinal cords at 8 weeks after SCI was performed (**Figure** **8**A). The CGCHF hydrogel combined with the NSCs treatment group showed an increase in Tuj1 positive cells due to the supplementation of exogenous NSCs. After combining with MP, Tuj1 positive cells further increased, indicating that improving the inflammatory inhibitory microenvironment was beneficial for the differentiation of NSCs. The CGCHF hydrogel loaded with MP and NSCs combined with the SMF treatment group had the most Tuj1 positive cells, indicating that external magnetic field regulation can promote more neuronal differentiation of NSCs (Figure 8B,D). Further exploring the role of SMF, immunofluorescence staining of NF200 and MBP was used to quantify axonal and myelin regeneration at the site of injury. In the case of SMF treatment, the intensity of NF200 positive axons was higher, and the NF200 positive axons tended to form new connections, indicating that the axon regeneration of NSCs could be guided under the magnetic field (Figure 8C,E). Meanwhile, the intensity of MBP positive cells was higher with SMF treatment, indicating more myelin regeneration (Figure 8C,E). In order to clarify the functional types of neurons differentiated from exogenous NSCs in vivo, immunofluorescence staining was performed on GABAergic neuron marker GAD‐1, cholinergic neuron marker ChAT, and 5‐hydroxytryptamine neuron marker 5‐HT. The results revealed an obvious enhancement in GAD‐1, ChAT, and 5‐HT positive cells in the SMF treatment group, indicating that exogenous NSCs were stimulated by SMF to differentiate more into GABAergic neurons, cholinergic neurons and 5‐hydroxytryptaminergic neurons. (Figure 8F,G,H). These findings demonstrated that under the regulation of SMF, exogenous NSCs after endocytosis of DMSA@Fe_3_O_4_ in vivo could differentiate more into neurons and promote axonal and myelin regeneration in the scaffold, together, increase the number of GABAergic neurons, cholinergic neurons, and 5‐hydroxytryptamine neurons (Figure 8A).

## 3 Discussion

SCI is a serious traumatic disease with devastating effect due to various pathological processes including inflammatory microenvironment, insufficient endogenous NSCs, difficulty in nerve regeneration, and the reconstruction of the neural circuit.^[^ ^1^ ^]^ Therefore, the present study develops a bifunctional NSCs delivery system based on a temperature‐responsive nanohydrogel that contains magnetic nanoparticles as the regulator of the physical mechanical stimulation and MP for the chemical drug therapy. It can repair SCI better by alleviating the inflammatory microenvironment, promoting the NSCs differentiation into neurons, and guiding the direction of axon regeneration. This multiple functional therapy is of great significance for clinical transformation, providing new treatment insights into SCI repair.

The delivery system is essentially a combined therapy for treating SCI. Based on the complex pathological processes of SCI, several targets have been discovered as potential therapeutic interventions including reduction of secondary damage, replacement of lost cells, removal of inhibitory factors, regenerative responses, resupply of neurotrophic support, remyelination of axons, rehabilitation strategies of neuroplasticity, and neuronal connections.^[^ ^18^ ^]^ Although individual therapy focusing on a singular barrier has been proven promising in preclinical SCI animal models, it only exerts modest and incomplete functional recovery, hindering their translation toward clinical trials.^[^ ^19^ ^]^ Recently, combinational strategies have been confirmed to have better beneficial outcomes than their individual treatment alone by aiming at the various pathological processes.^[^ ^20^ ^]^ In order to the combination of cellular transplants with reduction of the inhibitory factors, DePaul and his colleagues combined ChABC + acidified FGF intraspinal injection mixture with schwann cells, which showed that the combination enhanced robust axonal growth and functional improvements, while axons cannot grow into the graft without ChABC and aFGF.^[^ ^21^ ^]^ Fan et al. developed a collagen scaffold loaded with taxol and cetuximab targeting the intrinsic growth response, and the result showed that in the spinal cord completely T10 horizontal model, the implantation of the combined function scaffold has significantly increased the nerve regeneration to re‐connect the neural network and improve functional recovery.^[^ ^22^ ^]^ In order to inhibit the local inhibitory microenvironment to reduce secondary damage before cell transplantation, this study constructed nanohydrogel to sustained release MP and added HA to enhance the biocompatibility of NSCs, which could differentiate into various cells including neurons, astrocytes and oligodendrocytes to replace the lost cells. Meanwhile, neuronal regeneration and connections, myelin sheath regeneration, and axonal elongation were promoted after endocytosis of DMSA@Fe_3_O_4_ nanoparticles under the guidance of SMF. Altogether, this combination therapy produced a significant therapeutic effect on spinal cord complete transection injury in vivo. This multiple combination therapy has a certain potential for clinical transformation, but further validation is still needed in large animals such as beagles or crab‐eating monkeys.

Recent advancements in bioactive scaffold have involved the application of hydrogels as a promising delivery system for cells and small molecule drugs in repairing SCI.^[^ ^23^ ^]^ Hydrogels can mimic the matrix for cell growth, and achieve stable release of drugs and neurotrophin, thereby structurally and chemically supporting tissue regeneration and promoting axonal regrowth.^[^ ^24^ ^]^ The stimulus‐responsive hydrogel can induce different responses by many physical and chemical stimuli (temperature, electric, magnetic, pH,etc), which are very promising for developing drug delivery and regulating cell differentiation.^[^ ^25^ ^]^ Li and his colleagues created a polymer bioactive system composed of the thermosensitive hydrogel with umbilical cord mesenchymal stem cells, bFGF, ECM, and heparin‐poloxamer, and showed that it could reduce apoptosis and improve the mitochondrial functional recovery to produce good therapeutic effects in spinal cord contusion mouse model.^[^ ^26^ ^]^ Fan et al. developed an exosomes‐loaded electroconductive hydrogel that could synergistically inhibit inflammation and promote neuronal and axonal regeneration to better functional recovery in an SCI mouse model.^[^ ^27^ ^]^ Wang et al prepared a ferrofluid hydrogel with Fe_3_S_4_ and carboxymethyl chitosan which reduced the expression of inflammatory factors and promoted directional axonal regrowth and functional recovery after SCI.^[^ ^28^ ^]^ In this study, a temperature sensitive CS/β‐GP hydrogel system was established to achieve the injectable minimally invasive treatment. At the same time, CNCs were added to construct nano hydrogels to continuously release MP to reduce the inhibitory microenvironment at the acute inflammatory phase after SCI. In addition, magnetic particles (DMSA@Fe_3_O_4_) were added to construct magnetic hydrogels, which could be regulated by an external magnetic field after endocytosis of DMSA@Fe_3_O_4_ nanoparticles by NSCs and promoted NSCs into neurons and directional axonal growth.

NSCs are an attractive and promising cell source for the treatment of SCI, and they can differentiate into neurons, astrocytes and oligodendrocytes and secrete nutritional factors to improve the microenvironment.^[^ ^5^ ^]^ Promoting NSCs differentiation into more functional neurons, nerve regeneration, and circuit reconstruction has always been a crucial challenge for its application. With the advancement of nanoscience and technology, magnetic nanoparticles (MNPs) have come to the foreground result of the possibility of remote non‐invasive control by an external magnetic field.^[^ ^17^ ^]^ The inherent characteristics of MNPs enable them as remote actuators after being endocytosed by stem cells encapsulated in hydrogels by the external magnetic field.^[^ ^29^ ^]^ In an interesting research, iron oxide nanoparticles were used to develop a poly ‐L‐lactic acid electrospun fiber scaffolds arranged by magnetic response, which provided directional guidance for neurons.^[^ ^30^ ^]^ Also, a 3D magnetic hyaluronic hydrogel was designed with thiol‐functionalized magnetic microparticles and used for magnetomechanical modulation of primary DRGs neurons, showing the potential for future clinical applications, including remote non‐invasive neural stimulation and neuroregenerative medicine.^[^ ^31^ ^]^ In our study, NSCs internalized nanoparticles in hydrogel under SMF, differentiated more into functional neurons, promoted myelin regeneration, and inhibited astroglia, which were related to the activation of the PI3K/AKT/mTOR pathway. In vivo, the delivery system loaded with MP and NSCs combined with SMF treatment have been further confirmed to have a better therapeutic effect. However, there is still a controversy that needs further exploration regarding the intensity and duration of magnetic fields that regulate stem cell differentiation. Much literature on this issue is inconsistent due to the differences between cells and diseases, some using magnetic discs and others using instruments.^[^ ^32^ ^]^ The magnetic field device designed in this study could stably generate an SMF with uniform direction, producing satisfactory results but far from sufficient for clinical translation by the common parameters. Further research will be explored for the optimal work parameters in vivo and in vitro.

The severe inflammatory inhibitory microenvironment after SCI greatly hinders nerve cell survival, nerve regeneration, and functional recovery.^[^ ^10^ ^]^ Macrophages are more inclined to transform into the M1 phenotype rather than the M2 phenotype, which is more conducive to reducing inflammation and promoting tissue repair.^[^ ^33^ ^]^ It is widely believed that attenuating the inflammatory response and promoting macrophage M2 polarization during the acute phase of SCI is beneficial for SCI repair and the proliferation and survival of NSCs.^[^ ^34^ ^]^ MP, an anti‐inflammatory drug recommended for SCI, is controversial due to the high‐dose and long‐term systemic administration which can lead to serious complications.^[^ ^11^ ^]^ In recent years, many biomaterials have been used as carriers for the delivery of MP, achieving good therapeutic effects.^[^ ^11a^ ^]^ A hydrogel‐nanoparticle system composed of PLGA‐based nanoparticles and agarose hydrogel was reported to deliver MP which significantly decreased early inflammation by a significant reduction in the number of macrophages and a significantly diminished expression of iNOS in a spinal cord contusion model.^[^ ^35^ ^]^ The delivery system we designed can achieve local release of MP at first seven days during the acute phase of SCI, then exert an anti‐inflammatory effect, promote M2 polarization, and inhibit neuronal apoptosis.

This study inevitably has some limitations. The delivery system proposed in this study mainly targets the inhibition of inflammation and cell transplantation but does not cover more targets, which may limit the functional recovery effect after SCI. In addition, this study has proved the regulation of the NSCs differentiation and mechanism of magnetic field nanoparticles and verified their biocompatibility and cellular localization. However, further exploration was not conducted on the metabolism of nanoparticles in cells and mice. Finally, the magnetic field device designed in this study generated an SMF with uniform direction by the common parameters without exploring in detail the effects of different intensities and duration of magnetic fields on NSCs and SCI. Further research will be explored for the optimal work parameters in vivo and in vitro.

## 4 Conclusion

The present research develops a novel physico‐chemical bifunctional stem cells delivery system containing magnetic nanoparticles and methylprednisolone to achieve better functional recovery in SCI mice, which can alleviate the inflammatory microenvironment by promoting microglial M2 polarization, promote the NSCs differentiation into neurons and axon regeneration via the activation of the PI3K/AKT/mTOR pathway. This bifunctional NSCs delivery system combined physical mechanical stimulation and chemical drug therapy is demonstrated to be effective, which provides new treatment insights into clinical transformation of SCI repair.

## Conflict of Interest

The authors declare no conflict of interest.

## Author Contributions

W. Z., M. L., and J. R. contributed equally to this work. W. Z. designed and synthesized the delivery system under the guidance of Z.W., L.J., S.F. and X.S., M.L., X.Z., and J.R. performed the in vitro and in vivo experiments. With guidance from Z.W., L.J., S.F., and X.S., W.Z., and M.L. analyzed the data and wrote the manuscript. L.Y., S.H., D.Z., X.G, H.F., and B.Z. provided experimental guidance and conducted the experimental verification. Z.W., L.J., S.F., and X.S. provided guidance during all stages of the project. The manuscript was completed through contributions of all authors. All authors have given approval to the final version of the manuscript.

## Supporting information

Supporting Information

## Data Availability

The data that support the findings of this study are available on request from the corresponding author. The data are not publicly available due to privacy or ethical restrictions.
