# Supplementary material for: Magnetic Nanoparticles and Methylprednisolone Based Physico‐Chemical Bifunctional Neural Stem Cells Delivery System for Spinal Cord Injury Repair
Source: Adv Sci (Weinh). 2024 Mar 22;11(21):2308993. doi: 10.1002/advs.202308993 (PMC11151057; doi:10.1002/advs.202308993)
Supplement: Supplementary file 1 — Supporting Information [file ADVS-11-2308993-s001.pdf]

ADVANCED SCIENCE
Open Access

Supporting Information

for *Adv. Sci.*, DOI 10.1002/advs.202308993

Magnetic Nanoparticles and Methylprednisolone Based Physico-Chemical Bifunctional Neural Stem Cells Delivery System for Spinal Cord Injury Repair

Wencan Zhang, Mingshan Liu, Jie Ren, Shuwei Han, Xiaolong Zhou, Dapeng Zhang, Xianzheng Guo, Haiwen Feng, Lei Ye, Shiqing Feng, Xizi Song*, Lin Jin* and Zhijian Wei**

Supporting Information

Magnetic Nanoparticles and Methylprednisolone Based Physico-chemical Bifunctional Neural Stem Cells Delivery System for Spinal Cord Injury Repair

*Wencan Zhang, Mingshan Liu, Jie Ren, Shuwei Han, Xiaolong Zhou, Dapeng Zhang, Xianzheng Guo, Haiwen Feng, Lei Ye, Shiqing Feng,*Xizi Song,* Lin Jin,* and Zhijian Wei**

Supporting figures

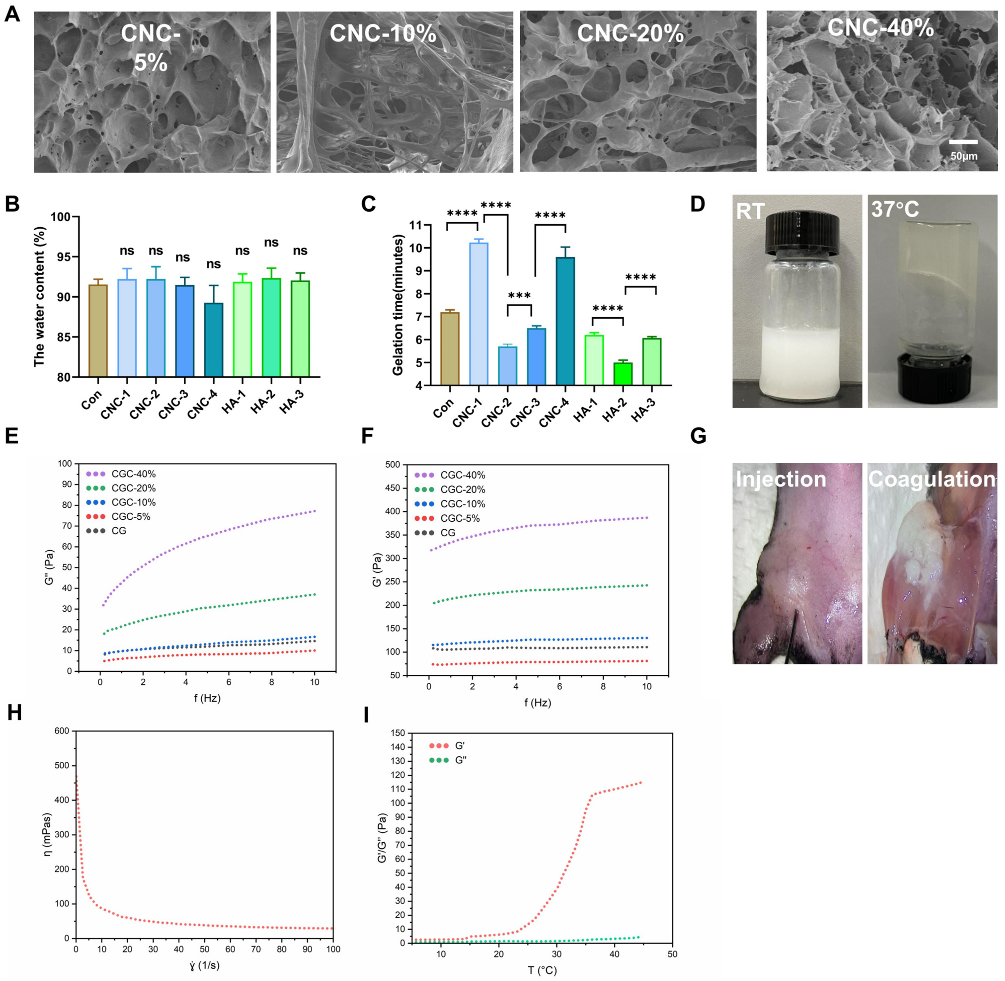

Figure S1. Exploration of the most suitable addition ratio of CNC and HA. A) SEM analysis showed that CGC hydrogel showed a 3D highly porous structure for cells to proliferation and differentiation. B, C) The gelation time and water content with different addition ratios of CNC and HA（n=3）. CNC-1/2/3/4 meant addition ratio of 5% CNC, 10% HA, 20% CNC and 40% CNC. HA-1/2/3/ meant addition ratio of 2% HA, 4% HA and 6% HA. D) CGCH hydrogel can be quickly gel at 37 ℃ in vitro. E, F) Rheological analysis showed that the storage moduli (elastic modulus, G′) and the loss moduli (viscous modulus, G″) of CGC hydrogel with 10% CNC was similar to that of CG hydrogel while reducing GP

concentration. G) CGCH hydrogel can be injected subcutaneously into C57 mice to form a hydrogel in situ. H) Rheological analysis showed that the shear thinning to establish the hydrogel's minimally invasive injection capability. I) Rheological analysis showed that the temperature-responsive of the CGCH hydrogel.

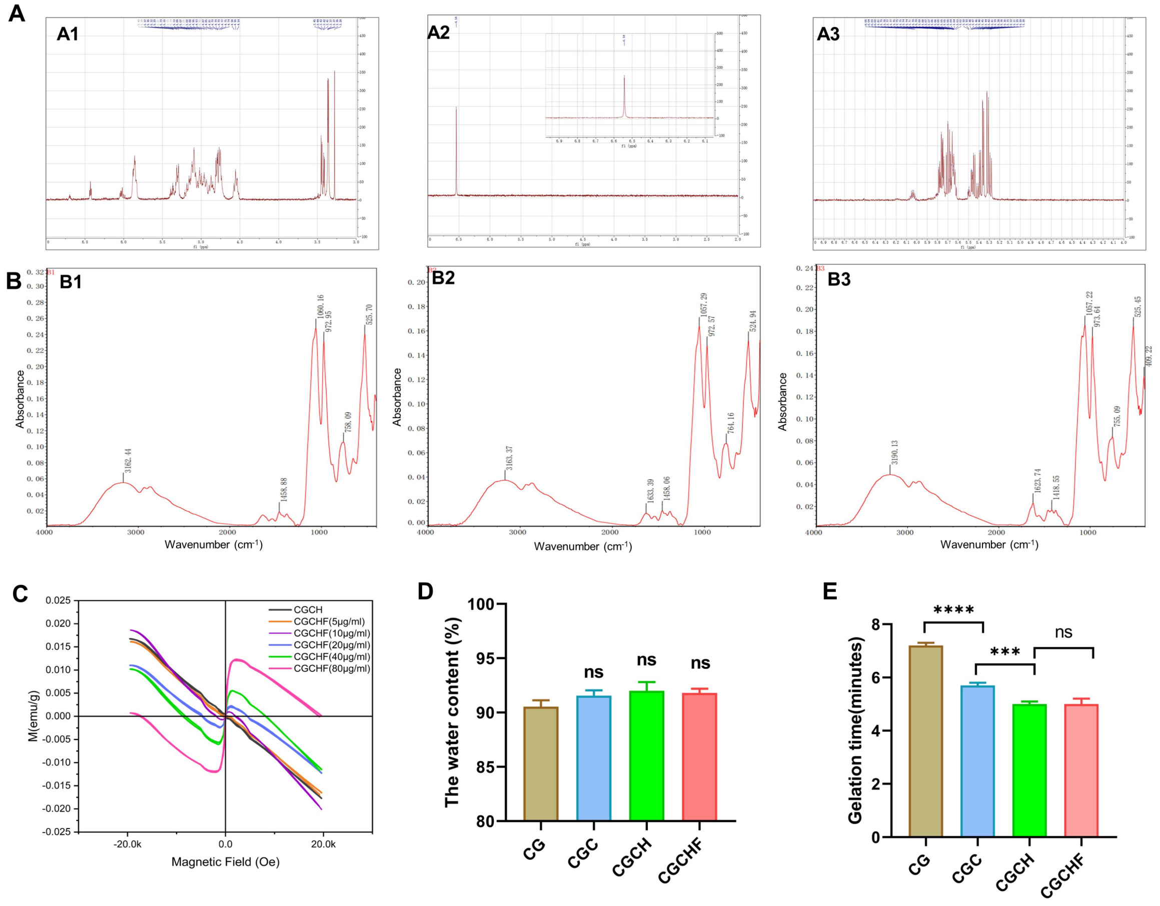

Figure S2. Characterization of CGCHF hydrogels. A) The result of 1H NMR of CNCs (A2) and 3.2~3.5ppm for HA (A1) based on the characteristic peak of CG hydrogel (A3). B) The results of FTIR of the CG hydrogel (B1), CGC hydrogel (B2) and CGCH hydrogel (B3). C) The result of the hysteresis loop indicated that the magnetic responsiveness of CGCHF hydrogels gradually increased as the concentration increased. D, E) The gelation time and water content of CG, CGC, CGCH and CGCHF hydrogels(n=3).

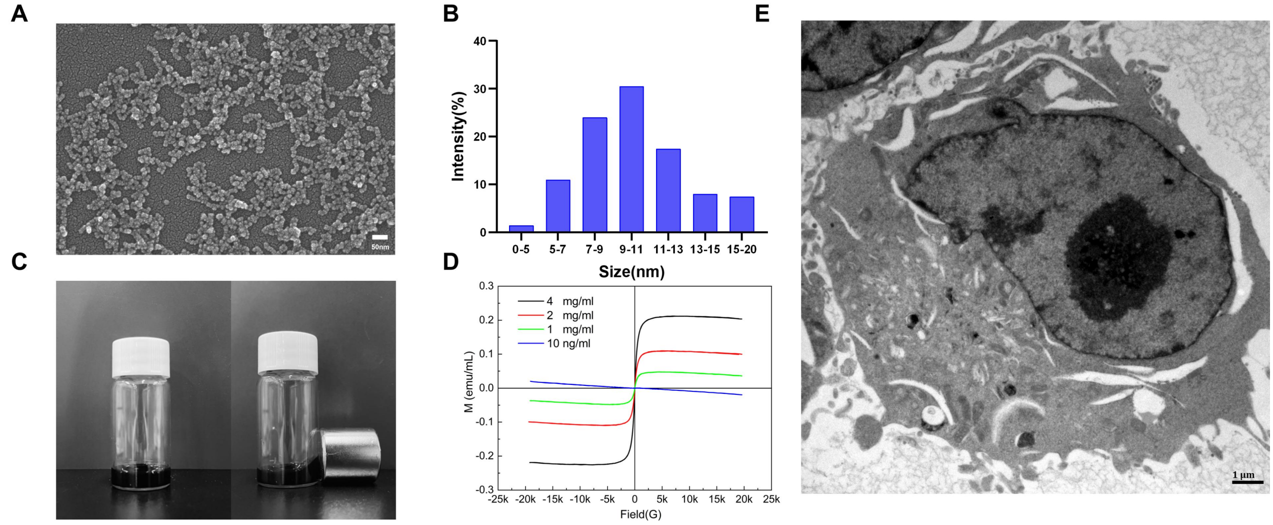

Figure S3. Characterization and optimal concentration of DMSA@Fe_3O_4. A, B) SEM analysis displayed the morphology of DMSA@Fe_3O_4 with the majority having a diameter of 10nm. Scale bar: 50 nm. C) DMSA@Fe_3O_4 can be attracted by a cylindrical magnet. D)The result of the hysteresis loop indicated that its magnetic responsiveness gradually increased as the concentration of DMSA@Fe_3O_4 increased. E) TEM images of NSCs. Scale bar: 1 μm.

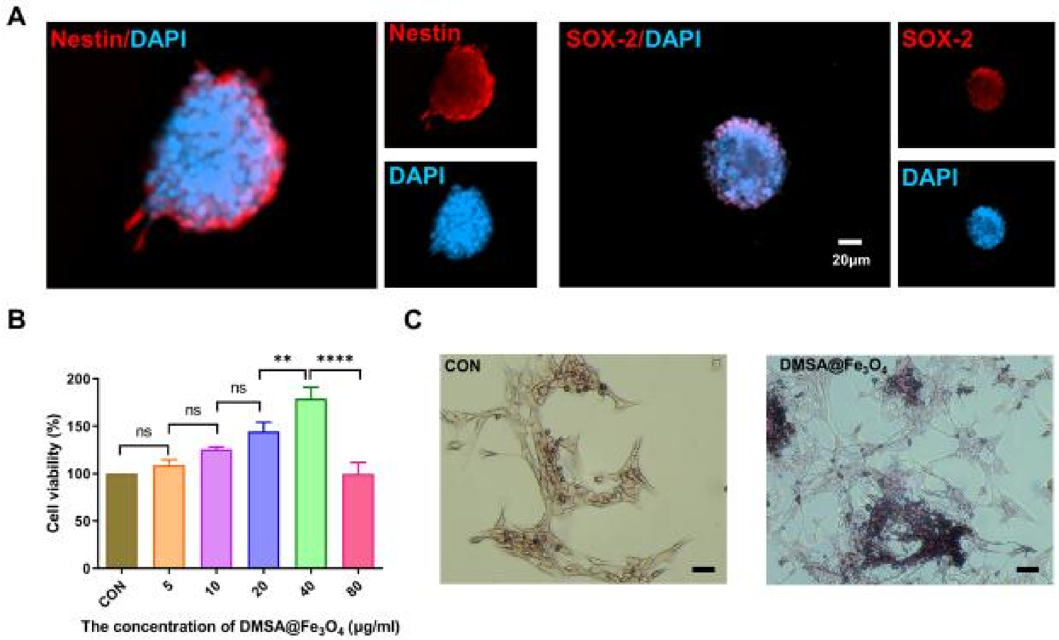

Figure S4. Identification of NSCs and toxicity of DMSA@Fe_3O_4. A) The immunofluorescence images of NSCs markers SOX2 and Nestin. Scale bar: 100 μm. B) The CCK8 results showed that DMSA@Fe_3O_4 has a certain dose-dependent effect on the proliferation of NSCs. However, the effect of promoting proliferation became weak when the concentration of DMSA@Fe3O4 reached 80ug mL^{-1} (n=3). C) Prussian blue staining results show that NSCs can absorb more DMSA@Fe_3O_4 at a concentration of 40ug mL^{-1}.Scale bar: 100 μm.

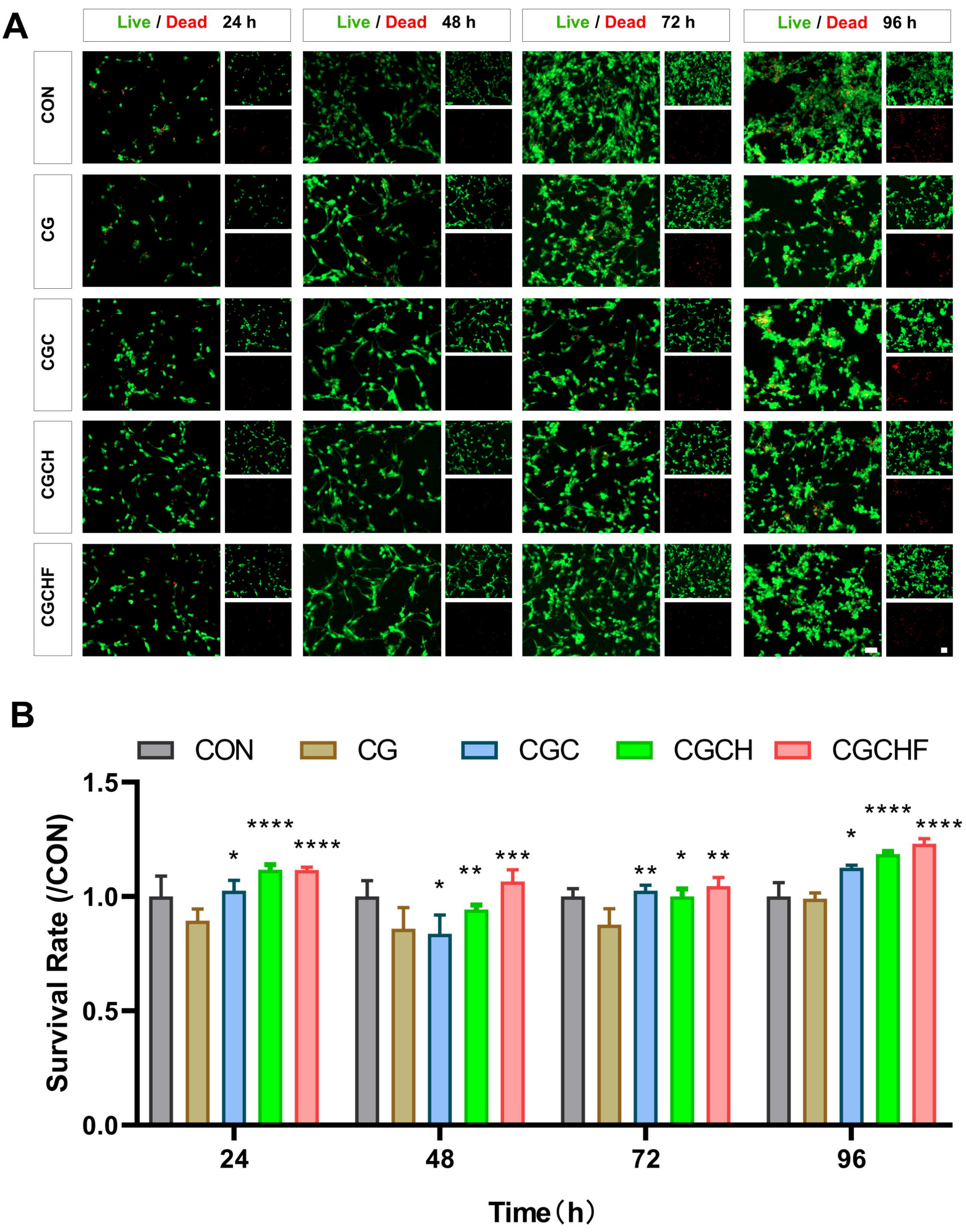

Figure S5. Biocompatibility of CG, CGC, CGCH and CGCHF hydrogels. A) The live/dead staining of NSCs after co-cultured with the hydrogels for 24/48/72/96 hours. Scale bar: 50 µm. B) Quantitative analysis of survival rate showed that the number of NSCs on the CG hydrogel decreased significantly at each time point when it was compared with the control group, while the number of NSCs on the CGC hydrogel increased, which was related to the lower β-GP concentration. After adding HA and DMSA@Fe_3O_4, the proliferation of NSC cells was further improved (n=3).

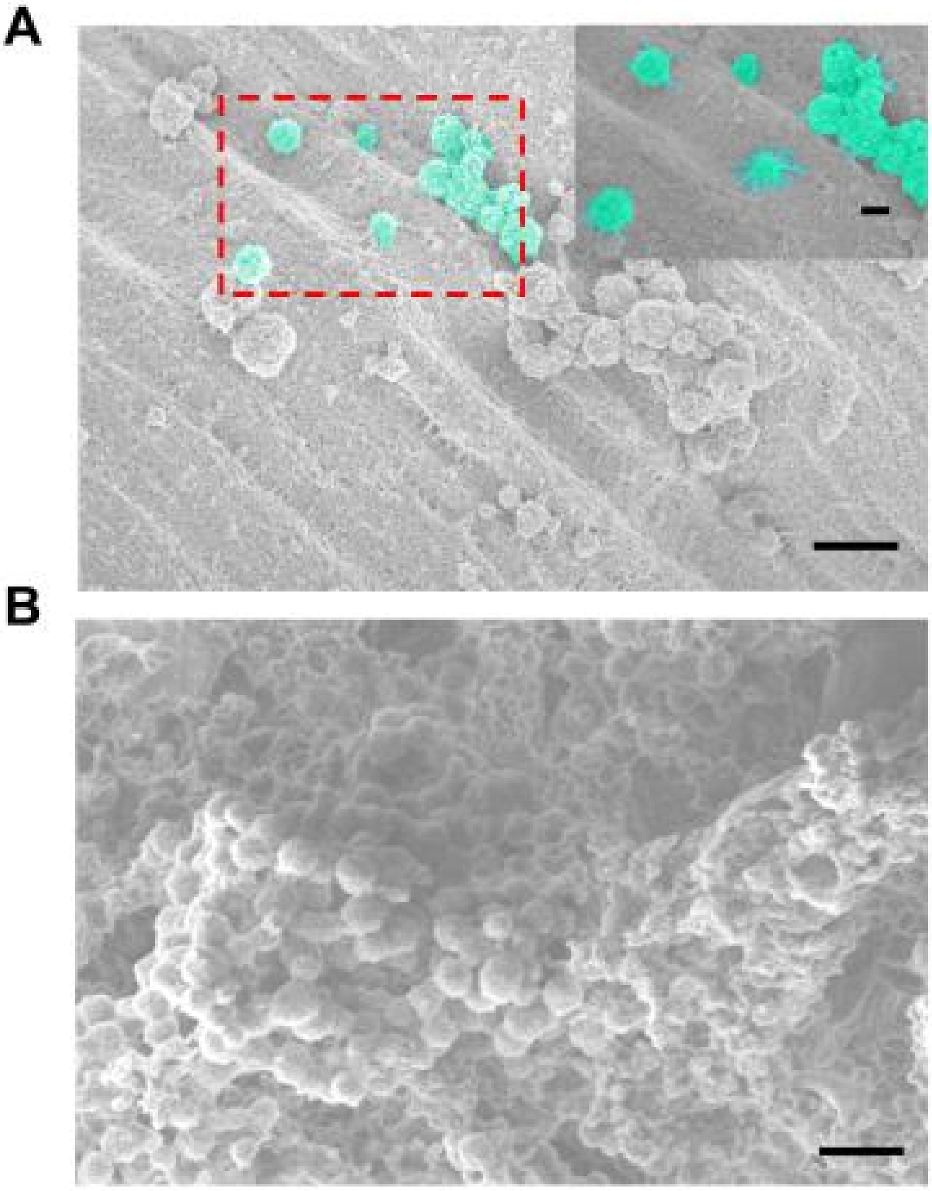

Figure S6. SEM images of NSCs onside/inside CGCHF hydrogels. A) SEM results showed that NSCs could adhere well on the surface of CGCHF hydrogel. Scale bar: 50 µm. The image in the upper right corner is an enlarged image in the red box. Scale bar: 10 µm. B) SEM results showed that NSCs could proliferate well inside CGCHF hydrogel.Scale bar: 50 µm.

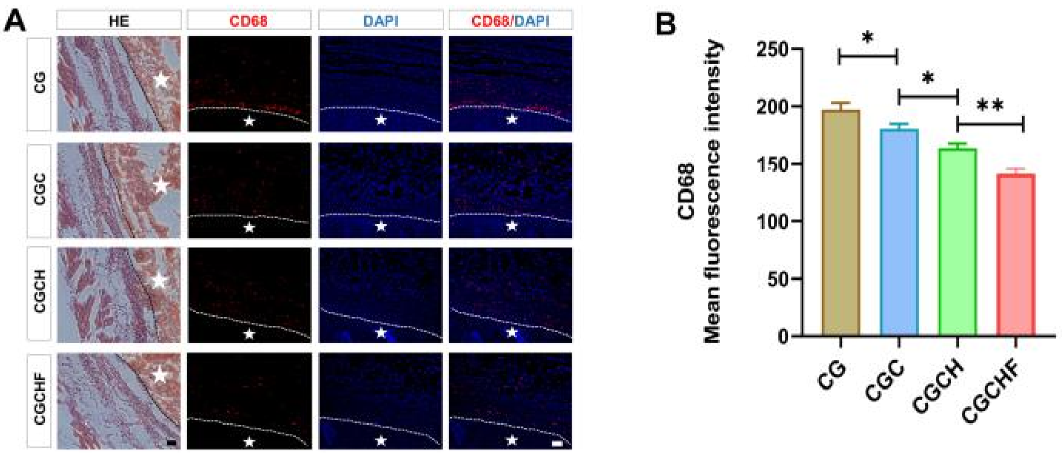

Figure S7. The subcutaneous inflammatory reaction of CG, CGC, CGCH and CGCHF hydrogels. A) HE staining and immunofluorescence of CD68 showed that one week after the implantation of the hydrogel, neutrophils infiltrated subcutaneously around the hydrogel, and the intensity of CD68 (macrophage marker) was lower in CGCHF hydrogels. The stars represented the hydrogel embedded subcutaneously. Scale bar: 100 μm. B) Quantitative analysis of the number of CD68 positive cells (n=3).

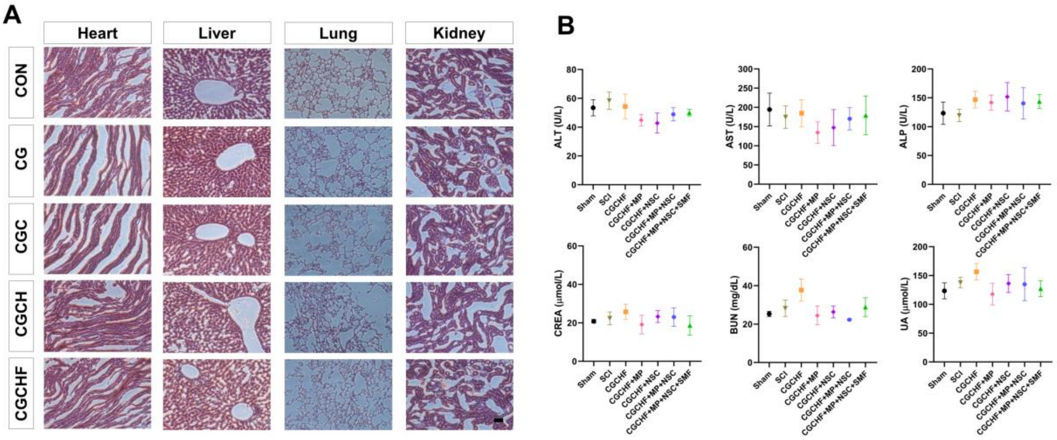

Figure S8. Biosafety of major organs after the implantation of the hydrogels. A) HE staining analysis confirmed that the main organs (heart, liver, lung, kidney) of mice treated with hydrogel in each group had no noticeable accumulation of hydrogel degradation products and no obvious pathological abnormalities. Scale bar: 100 μm. B) In vivo animal experiments have shown that the levels of important liver and kidney function indicators such as ALT, AST, ALP, CERA, BUN and UA in SCI mice treated with different treatments were within the normal range indicating that the multiple combination therapy proposed in this study did not cause systemic toxicity in SCI mice (n=3).

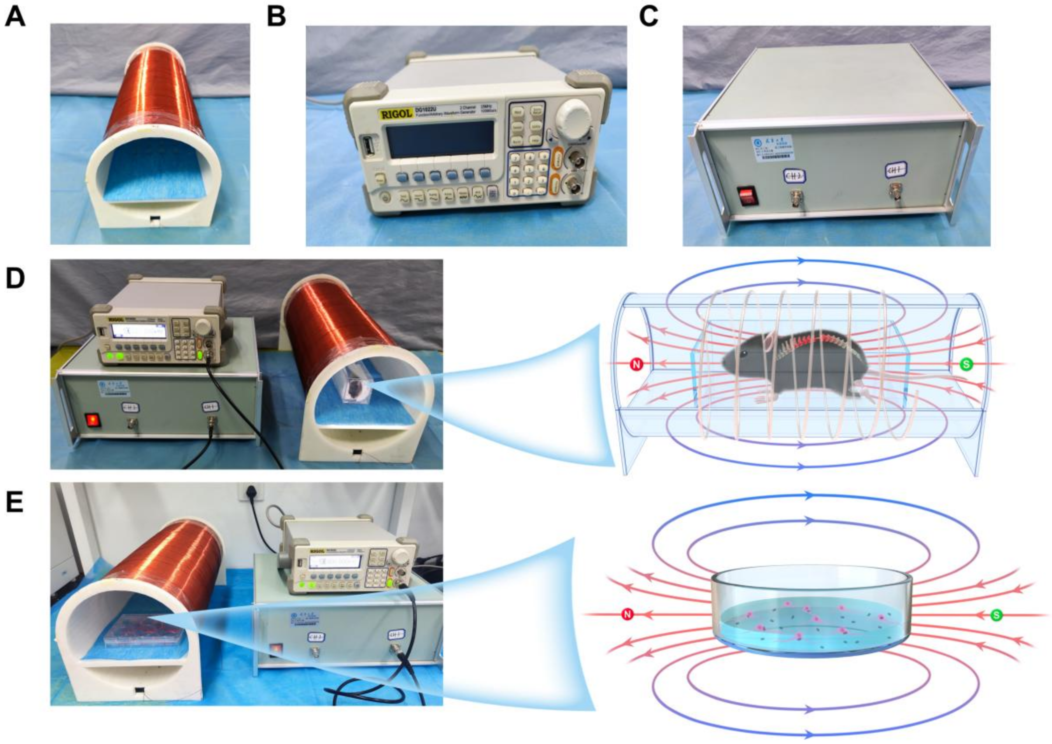

Figure S9. Design of Static Magnetic Field Instruments. A) The excitation coil was composed of a hollow cylinder by 3D printing and a copper coil around the outside of the cylinder. B) The signal generator. B) The power amplifier. D, E) According to adjusting the parameters of the signal generator, a stable static magnetic field can be generated at the center of the coil, with the magnetic field line parallel to the axis of the cylinder and passing longitudinally through the NSCs in the cell culture plate and the spinal cord of mice. The parameters of the signal generator in this study are sine wave, frequency 50Hz, and amplitude 2.2V. After fixed amplification by the power amplifier (with a magnification of about 36 times), the magnetic field intensity generated at the center of the coil is about 1mT.

Table S1. The primer sequences used in these experiments.

Table S1. The primer sequences used in these experiments.

Geen name	Forward (5' to 3')	Reverse (5' to 3')
β-actin	GGCTGTATTCCCCTCCATCG	CCAGTTGGTAACAATGCCATGT
Nestin	TGCCCTAGAGACGGTGTCTCA	AATCGCTTGACCTTCCTCCC
Tuj-1	TATGAAGATGATGACGAGGAATCG	TACAGAGGTGGCTAAAATGGGG
MAP-2	GCCAGCCTCAGAACAAACAG	AAGGTCTTGGGAGGGAAGAAC
GFAP	CCAAGCCAAACACGAAGCTAA	CATTTGCCGCTCTAGGGACTC
iNOS	CCTGCTTTGTGCGAAGTGTC	CCCAAACACCAAGCTCATGC
IL-12	AGTGACATGTGGAATGGCGT	CAGTTCAATGGGCAGGGTCT
TNF-α	AGCCGATGGGTTGTACCTTG	ATAGCAAATCGGCTGACGGT
IL-1β	TGCCACCTTTTGACAGTGATG	TGATGTGCTGCTGCGAGATT
Arg-1	TGTCCCTAATGACAGCTCCTT	GCATCCACCCAAATGACACAT
TGF-β	AGCTGCGCTTGCAGAGATTA	AGCCCTGTATTCCGTCTCCT

5. Experimental Section

Preparation of Various Components in Hydrogel.

Chitosan (CS) powder (C804726, Mackin, Shanghai, China) and β-Glycerol phosphate disodium salt (β-GP) powder (G806967, Mackin, Shanghai, China) was spread and irradiated with ultraviolet light for 24 hours. Then, CS powder was dissolved to form a 2% CS solution in a 0.1 M sterile hydrochloric acid solution and stored at 4°C. β-GP powder was dissolved to form a 30% and 50% (w/w) β-GP solution in sterile ddH_2O and storage at 4°C. CS/β-GP (CG) hydrogel is temperature sensitive which can be injected into the body and form a gel at 37 °C to fill the damaged part. Meanwhile, it has an advantage in drug controlled release and seed cell encapsulation. However, the speed of the gel depends on the concentration of β-GP, which is biologically toxic at high concentrations.

Cellulose nanocrystals (CNCs) dispersion (Qihong, Guilin, China)was used as a structural stabilizer of hydrogels with a content of 5.6%, a pH of 7, a fiber diameter of 5~10nm and a length of 100~500um, followed by high-pressure sterilization and storage at 4°C. When mixed with CS/β-GP hydrogel, it can reduce the concentration of β-GP and enhance the biocompatibility of hydrogel.

Hyaluronic acid (HA) crystal (H131007, Aladdin, Shanghai, China) dissolved in 30% β-GP solution to form a 2%, 4% and 6% HA/β-GP solution and storage at 4°C. As a structural component of hydrogel that can promote the attachment of seed cells, HA can improve the biocompatibility of hydrogel.

DMSA coated Fe_3O_4 nanoparticles (DMSA@Fe_3O_4, Zhongkeleiming, Beijing, China) were dispersed in ddH_2O with 4mg/mL, followed by high-pressure sterilization and storage at 4°C. DMSA@Fe_3O_4 has magnetic responsiveness, which can be regulated by an external magnetic field as a component of the hydrogel. At the same time, it has superparamagnetism, which can avoid the magnetocaloric effect in the static magnetic field.

Synthesis of Hydrogel.

The 50% β-GP solution was added to the 2% CS solution at a volume ratio of 1:5 to form the CG hydrogel. To reduce the concentration of β-GP, CNCs were added at a different volume ratio of 5%, 10%, 20%, or 40% with 30% β-GP solution to form the CS/β-GP/CNCs (CGC) nanohydrogel. After the optimal CNCs ratio was determined, HA was added to enhance biocompatibility. The 2%, 4% and 6% HA/β-GP solution were added into the 2% CS with solution optimal CNCs ratio to obtain the CS/β-GP/CNCs/HA (CGCH) nanohydrogel. Finally, DMSA@Fe_3O_4 nanoparticles were mixed with the CGCH solution to obtain CS/β-GP/CNCs/HA/DMSA@Fe_3O_4 (CGCHF) nanohydrogel. The final concentration of DMSA@Fe_3O_4 nanoparticles (40μg/mL) was determined by their biocompatibility and magnetic responsiveness.

Characterization of Hydrogel Properties.

Gelation Time: The mixed solution of hydrogels was obtained with various components at different volume ratios. The coagulation time of hydrogel was calculated from the beginning of adding β-GP or HA/β-GP solution in a 37°C water bath until the hydrogel mixed solution turned into a solid and could not flow after the test tube bottle was inverted.

Water Content and Swelling Property: The weight of hydrogel (W0) was recorded after solidification. Then the dried weight (W_1) was recorded after the freeze-drying method under a vacuum at −80°C for 24 h. The water content was measured by the formula: water content= $(W_0-W_1)/W_0$. Scanning Electron Microscope (SEM): After the hydrogels were freeze-dried, liquid nitrogen was used to break the hydrogels to maintain their original internal cross-linking structure. Then Aurum was sputtered to coat the hydrogels. The morphology was observed by SEM (ZEISS GeminiSEM 300, Germany) under 10kV accelerating voltage.

1H NMR of hydrogel: The chemical modification of hydrogel was evaluated by proton nuclear magnetic resonance (1H NMR) spectrum. After CG, CGC, and CGCH were dissolved

in deuterated hydrochloric acid, the 1H NMR spectra of hydrogels was tested by the Bruker spectrometer at a 1H resonance frequency of 400MHz.

FTIR (Fourier transform infrared spectroscopy): The FTIR spectra of CG, CGC and CGCH hydrogels were detected by Thermo Scientific Nicolet iS5 spectrometer. After being freeze-dried, the hydrogels were broken by liquid nitrogen, then ground into powder to make the hydrogel become particles, and finally recorded by spectral software.

Rheological experiment: The rheological properties were measured with the rotary Rheometer (Haake Mars60, Germany). Frequency scanning (0.1-10Hz) was performed under a fixed strain of 1%. Hydrogels were placed onto the parallel plate and preheated to 37°C to solidify it, then lowered the upper plate to 1mm gap for detection.

Magnetic hysteresis loop experiment: The magnetic nanoparticles were mixed into the hydrogels with different concentrations. Then, the hydrogels were frozen and dried after being solidified into a gel and grounded into powder. Then the magnetic hysteresis loop of the samples was tested by vibrating sample magnetometer (VSM, LakeShore7404) ranging from -2T to +2T at room temperature.

MP release using high-performance liquid chromatography: Mix the MP evenly into the hydrogel, put it into the dialysis bag after turning it into gel at 37°C, then immerse it in the test tube bottle containing ddH_2O, and then vibrate continuously in 37°C incubator. Collect the liquid in the bottle daily, record the volume V_1 until 14 days, and replace it with new ddH_2O. Afterwards, the content of MP of each sample was determined using high-performance liquid chromatography (Prominence LC-20A, Japan) and record the concentration C_1. The cumulative release of MP was calculated using the following formula: cumulative release= $\sum_{i=14}^{1} ViCi$.

Isolation of Neural Stem Cells (NSCs) and BV2 Cell Culture.

NSCs were extracted from the cerebral cortex of the embryos of C57/BL6 female mice on day 14 of pregnancy in a sterile environment, as described previously.[1] Briefly, the cerebral cortex of embryos was dissected. It was cut into pieces and digested with DNase I (Sigma, the US) and Papain (Worthington Biochemical, the US) for 10 minutes and obtained single cell suspension, which was then incubated with the culture medium at 37°C with 5% CO2. NSCs were cultured in proliferation medium: Neurobasal medium (Gibco, the US) supplemented with 2% B-27 supplement without vitamin A (Gibco, the US), 1% glutamineMAXTM^{-1} (Gibco, the US), 20 ng mL^{-1} bFGF (Peprotech, the US), 20 mg mL^{-1} EGF (Peprotech, the US) and 1% penicillin/Streptomycin (Gibco, the US). Normal NSCs can form neurospheres after proliferation, which can be observed under light microscopy. Meanwhile, the surface antibodies such as Nestin and Sox2 were verified using immunofluorescence. Cells from the 3rd to 5th generations were used for experiments. The BV2 cells were cultured in DMEM-HG (Gibco, the US) with 10% FBS and 1% penicillin/Streptomycin.

NSC Viability, Proliferation on the Hydrogel.

Each hydrogel was immersed in the neurobasal medium (1g: 50ml) for 24 hours in the 37°C incubator to obtain the extract, which was used to prepare the proliferation medium as described above. Then NSCs were cultured by the above proliferation medium with the extract. Live/Dead Staining and CCK8 analysis at 24/48/72/96 hours were performed to assess the viability of NSCs. The cells were incubated with calcein-AM and PI (Beyotime, China) for 30 minutes. A confocal laser scanning microscope (CLSM, Zeiss, Germany) was used to observe cells. The CCK-8 (Beyotime, China) solution was added to the cells at a ratio of 1:10. After incubation for two h, the mixed solution was detected with an enzyme-labeling instrument (Thermofisher, the US). NSCs were encapsulated into hydrogels cultured with proliferation medium for 48h and visualized by CLSM to analyze the three-dimensional proliferation of cells by Live/Dead Staining. SEM was used to observe cell proliferation in the hydrogel.

Design of Static Magnetic Field Instruments.

In order to enable cells to receive stable static magnetic field regulation after endocytosis of nanoparticles, as well as for physical therapy in animal experiments, we have designed devices that can generate magnetic fields, as shown in Figure S9. Firstly, we used 3D printing to create a hollow cylinder with a platform inside that can accommodate cell culture plates and mice. Next, we uniformly wrap the copper coil around the outside of the cylinder to form an excitation coil (Figure S9A, Supporting Information). Finally, the excitation coil is connected with the signal generator and power amplifier to form the magnetic field device(Figure S9B, C, Supporting Information). According to adjusting the parameters of the signal generator, a stable static magnetic field can be generated at the center of the coil, with the magnetic field line parallel to the axis of the cylinder and passing longitudinally through the NSCs in the cell culture plate and the spinal cord of mice(Figure S9D, E, Supporting Information). The parameters of the signal generator in this study are sine wave, frequency 50Hz, and amplitude 2.2V. After fixed amplification by the power amplifier (with a magnification of about 36 times), the magnetic field intensity generated at the center of the coil is about 1mT.

NSCs Viability with DMSA@Fe_3O_4 and the Position of MNPs.

Different concentrations of DMSA@Fe_3O_4 nanoparticles were added to NSCs in a proliferation culture medium, and the viability of NSCs was observed at 48 hours. Based on the result of CCK8 and magnetic responsiveness, the optimal concentration of nanoparticles in the hydrogel was determined. In order to explore the position of nanoparticles in NSC proliferation and differentiation, nanoparticles were labeled with FITC, which can exhibit green light under 488nm laser irradiation. NSCs were cultured with DMSA@Fe_3O_4 nanoparticles in a proliferation culture medium, and then glutaraldehyde solution and osmic acid solution were used to fix the cells. The sample was processed through alcohol gradient dehydration and acetone solution. An ultramicrotome (Leica UC7) was used to obtain ultrathin sections. TEM (Hitachi H-7650) was used to observe the samples after staining.

NSCs Differentiation.

NSCs were cultured on a plate coated with 0.01% Poly-L-Lysine solution (Sigma, the US)in differentiation medium, whose formulation was DMEM/F12 (Gibco, the US) supplemented with 2% B-27 supplement without vitamin A, 1% FBS, 1% glutamineMAXTM^{-1} and 1% penicillin/Streptomycin. Then, DMSA@Fe_3O_4 nanoparticles were added to the culture well after cell adhesion. The culture well with nanoparticles was placed in the static magnetic field for 2 hours daily. After seven days, cells were collected for RNA-seq, q-PCR or WB and fixed with 4% paraformaldehyde for immunofluorescence.

Co-culture System of BV2 Cells and Hydrogel.

BV2 cells were planted in the lower layer of the 6-well transwell chamber, and the lipopolysaccharides (LPS, 200ng/mL, Solarbio, China) were added for 24 hours to induce an inflammatory model. Hydrogels loaded with MP (50μg mL^{-1}) were placed onto the upper layer of the transwell chamber and immersed in culture mediumo to mimic sustained drug release for treatment.[2] After 24 hours, cells were collected for q-PCR or WB and fixed with 4% paraformaldehyde for immunofluorescence.

Primary Neurons Isolation, Culture and the OGD/R Model.

The steps for obtaining primary neurons and neural stem cells are basically the same as described previously.[38] After single cell suspension from the cerebral cortex of embryos was obtained, it was cultured on cell dishes coated by poly-L- lysine (Sigma–Aldrich, the US) with the culture medium: DMEM-HG with 10% FBS and 1% penicillin/Streptomycin. Then, the medium was replaced with Neurobasal medium supplemented with 2% B-27 supplement without vitamin A and 1% penicillin/Streptomycin.

After seven days, primary neurons were used for the OGD/R model. The OGD/R model was established using a previously described method.[3] After the culture medium was replaced with glucose-free Dulbecco's modified Eagle's medium (Gibco, the US), the cells were

cultured under hypoxic conditions for 1 hour to achieve OGD in GENbag anaerobic incubation system (bioMérieux SA, France). Then, the cells were cultured in a normal serum-free medium (OGD group) and serum-free medium with CGCHF hydrogel loading MP (MP group) under normal incubation conditions for 24 hours.

Gene Expression.

q-PCR: BV2 and NSCs were suspended in TRIzol (Invitrogen, the US), and a total RNA extraction kit (Solarbio, China) was used to harvest RNA. The RNA amount and purity were quantified by NanoDrop ND-1000 (NanoDrop, the US). Subsequently, the PrimeScript™ RT reagent kit (Takara, Japan) was used to reverse the mRNA into cDNAs. Afterward, Real-Time Quantitative Polymerase Chain Reaction (RT-qPCR) was performed using LightCycler 960 with TB Green® Premix Ex Taq™ II (Tli RNaseH Plus) (Takara, Japan). The primer sequences used in these experiments are shown in Table S1, Supporting Information.

RNA Sequence Analysis: Total RNA was isolated and purified using TRIzol (Invitrogen, the US) after NSCs were co-cultured with DMSA@Fe_3O_4 nanoparticles in the SMF. RNA sequence analysis was performed by LC-Bio Technology. Kyoto Encyclopedia of Genes and Genomes (KEGG) and Gene Ontology (GO) analyses were performed according to the differentially expressed genes between Con and DMSA@Fe_3O_4+SMF.

Western Blot.

BV2 and NSCs were lysed in RIPA lysis buffer (Beyotime, China) containing protease inhibitor (Beyotime, China) and phosphatase inhibitor (Beyotime, China). A BCA protein assay kit (Solarbio, China) was used to measure the protein concentration. Each protein suspension after denaturation by SDS loading buffer was separated on a 10% or 6% SDS-PAGE gel (Beyotime, China) and then transferred onto 0.22μm or 0.45 μm polyvinylidene fluoride membranes (PVDF, Invitrogen, the US). Then, the PVDF membranes were blocked with 5% BSA (bovine serum albumin, Sigma, Germany) for 2 hours and incubated with primary antibodies at four °C overnight. Secondary antibodies were added for 1 hour. An ultra-sensitive enhanced chemiluminescent (Invitrogen, the US) was used for protein detection by the ChemiDoc MP Imaging System (Bio-Rad, the US). The protein blot analysis was performed using Image J software. The primary antibodies used included: iNOS (22226-1-AP, Proteintech, the US), Arg-1 (66129-1-Ig, Proteintech, the US), Cleaved-caspase 3 (9661S, CST, England), Bax (ab182733, Abcam, England), Bcl-2 (ab32124, Abcam, England), β-actin (HRP-60008, Proteintech, the US), Nestin (19483-1-AP, Proteintech, the US), GFAP (ab4648, Abcam, England), Tuj1 (ab215037, Abcam, England), MAP2 (67015-1-Ig, Proteintech, the US), GAPDH (60004-1-Ig, Proteintech, the US). The secondary antibodies used included: HRP-conjugated Affinipure Goat Anti-Mouse IgG(H+L) (SA00001-1, Proteintech, the US), HRP-conjugated Affinipure Goat Anti-Rabbit IgG(H+L) (SA00001-2, Proteintech, the US).

Immunofluorescence Staining in Vitro.

The 4% PFA (paraformaldehyde, Solarbio, China) was used to fix the cells for 30 min. Then, the cells were blocked and permeabilizated) for 1 hour by 5% BSA with 0.3% Triton X-100 (Sigma, Germany. The samples were incubated with primary antibodies at four °C overnight and secondary antibodies for two hopur. Finally, the cells were stained with DAPI (Solarbio, China) for 10 min. CLSM was used to observe all samples. The primary antibodies included: Nestin (19483-1-AP, Proteintech, the US), SOX2 (66411-1-Ig, Proteintech, the US), Tuj1 (ab215037, Abcam, England), GFAP (3670s, CST, the US), and MAP2 (67015-1-Ig, Proteintech, the US), CD68 (ab125212, Abcam, England), iNOS (22226-1-AP, Proteintech, the US), Arg-1 (66129-1-Ig, Proteintech, the US), GFAP (ab53554, Abcam, England). The secondary antibodies included: CoraLite488-conjugated Goat Anti-Mouse IgG(H+L) (SA00013-1, Proteintech, the US), CoraLite594-conjugated Goat Anti-Rabbit IgG(H+L) (SA00013-4, Proteintech, the US), Donkey Anti-Goat IgG H&L (Cy3 ®) (ab6949, Abcam, England).

Tunel Staining of Neurons in Vitro.

The 4% PFA was used to fix the primary neurons for 30 min. Then, the cells were permeabilizated for 5 minutes by 0.3% Triton X-100. Then, the samples were incubated with Tunel working solution (Beyotime, China) in the dark at 37 º C for 60 minutes. After being stained with DAPI for 10 min, the cells were observed with a CLSM.

Ethics Statement of Animal Experiments.

All adult female C57BL/6J mice (6–8 weeks old) were supplied by Beijing Vital River Laboratory Animal Technology Co., Ltd. All animal experiments were approved by the Laboratory Animal Ethical and Welfare Committee of Shandong University Cheeloo College of Medicine (Jinan, China, Approval Number:22052).

Degradation and Biocompatibility of Hydrogel in Vivo.

Adult female mice (n=18) were used to assess the biodegradability and biocompatibility of the scaffolds in vivo. The mice were divided into six groups according to the sacrificed time at weeks 1, 2, 3, 4, 6, and 8 after scaffolds were injected subcutaneously into the back of the mice. The surrounding tissue with scaffolds was removed, then it was fixed in 4% paraformaldehyde for HE staining. The thickness of the fibrotic capsule and neutrophils were used to evaluate inflammatory response by Image J software.

Spinal Cord Complete Transection Model.

In order to verify the anti-inflammatory effect, adult female mice (n=24) were randomly assigned to four groups: sham, SCI, CGCHF, and CGCHF+MP (n=6). In order to figure out the effect of scaffolds in repairing SCI, adult female mice (n=42) were randomly assigned to seven groups: sham, SCI, CGCHF, CGCHF+MP, CGCHF+NSCs, CGCHF+MP+NSCs, CGCHF+MP+NSCs+SMF (n=6). Mice were anesthetized by isoflurane with a multi-channel small animal anesthesia machine (RWD Life Science Co., Ltd, China). At the T10 level, spinal cord complete transection with a 2 mm long longitudinal defect was performed, Where the hydrogels of 8 μL were injected into. Cell density in the hydrogel is one × 10^8 NSCs mL^{-1}. Nursing mice twice a day to manually empty their bladder.

Histological Analysis

Mice were deeply euthanized using isoflurane eight weeks post-SCI and were perfused with PBS and 4% PFA. The spinal cord was dissected and then fixed in 4% PFA for 24 h before being soaked in 20% and 30% sucrose solution for dehydration. Then the spinal cord was then cut into slices for immunofluorescence and HE staining.

Immunofluorescence Staining in Vivo

The tissue slices of 10μm were obtained by a frozen slicer (Leica, Germany). The 4% PFA was used to fix for 15 min. Then, the slices were blocked by 5% NGS (normal goat serum, Gibco, the US) and 5% BSA mixed with 0.3% Triton X-100 for 1 hour. The slices were incubated with primary antibodies at four °C overnight and secondary antibodies for 2 hours and were covered with mounting medium, antifading (with DAPI) (Solarbio China). All slices were observed by CLSM. The primary antibodies included: CD68 (ab125212, Abcam, England), Ibal-1(ab178846, Abcam, England), iNOS(ab210823, Abcam, England), Arg-1 (66129-1-Ig, Proteintech, the US), GFAP (ab53554, Abcam, England), NF200 (ab8135, Abcam, England), Laminin (ab11575, Abcam, England), Tuj1(ab78078, Abcam, England), MBP(78896S, CST, the US), Syn (ab32127, Abcam, England), GAD-1(67648-1-Ig, Proteintech, the US), Chat (ab178850, Abcam, England), $5HT^+$ (ab271031, Abcam, England). The secondary antibodies included: CoraLite488-conjugated Goat Anti-Mouse IgG(H+L) (SA00013-1, Proteintech, the US), CoraLite594-conjugated Goat Anti-Rabbit IgG(H+L) (SA00013-4, Proteintech, the US), Donkey Anti-Goat IgG H&L (Cy3 ®) (ab6949, Abcam, England), Anti-Rabbit IgG H&L (Alexa Fluor® 405) (ab175651, Abcam, England).

HE Staining.

The tissue slices were stained with hematoxylin solution (Solarbio China) for 2 minutes and the eosin solution (Solarbio China) for 60 seconds. Then, slices were soaked with ethanol for

gradient dehydration (75%, 85%, 95%, 100%) and xylene twice for 1 minute each time for transparency. Finally, the slices were covered with neutral gum (Solarbio China). All slices were observed by optical microscope.

Behavioral Functional Recovery Analysis.

BMS scores were used to calculate the degree of recovery of the hind limb behavior. The scores were recorded blindly by two observers ranging from 0 (no ankle movement) to 9 (complete function recovery). The bladders of mice were manually emptied twice daily and then were cut into slices for HE staining after sacrifice to assess the recovery of the bladder based on the thickness of the bladder wall. Motor evoked potential (MEPs) was measured by electrophysiological devices (BL420N, Techman, China) after mice were anesthetized with isoflurane at eight weeks post-SCI. A 10-mA current was used to constantly stimulate with a duration of 1 ms.

Statistical Analysis.

All values are represented as the mean ± standard deviation (SD). Statistical analyses were performed with GraphPad Prism 8.0 software and Origin 2022. Image J software was used to analyze the protein expression and fluorescence intensity. Statistical significance was calculated by one-way or two-way ANOVA followed by Bonferroni's comparison test. $p < 0.05$ were considered significant and the level of significant difference was defined as: *$p < 0.05$, **$p < 0.01$, ***$p < 0.001$, ****$p < 0.0001$.

References

[1] M. Hao, D. Zhang, W. Wang, Z. Wei, J. Duan, J. He, X. Kong, Y. Sang, S. Feng, H. Liu, *Adv. Funct. Mater.* **2022**, 2203492.

[2] Yang, BM. Conley, SR. Cerqueira, T. Pongkulapa, S. Wang, JK. Lee, KB. Lee. *Adv. Mater.* **2020**, e2002578.

[3] C. Zhang, X. Yi, M. Hou, Q. Li, X. Li, L. Lu, E. Qi, M. Wu, L. Qi, H. Jian, Z. Qi, Y. Lv, X. Kong, M. Bi, S. Feng, H. Zhou, *Elife* **2023**, 12, e85324.
